# Nuanced role for dendritic cell intrinsic IRE1 RNase in the regulation of antitumor adaptive immunity

**DOI:** 10.3389/fimmu.2023.1209588

**Published:** 2023-06-06

**Authors:** Felipe Flores-Santibañez, Sofie Rennen, Dominique Fernández, Clint De Nolf, Evelien Van De Velde, Sandra Gaete González, Camila Fuentes, Carolina Moreno, Diego Figueroa, Álvaro Lladser, Takao Iwawaki, María Rosa Bono, Sophie Janssens, Fabiola Osorio

**Affiliations:** ^1^Laboratory of Immunology and Cellular Stress, Immunology Program, Institute of Biomedical Sciences, Faculty of Medicine, University of Chile, Santiago, Chile; ^2^Immunology Laboratory, Biology Department, Faculty of Sciences, University of Chile, Santiago, Chile; ^3^Laboratory for ER Stress and Inflammation, VIB Center for Inflammation Research, Ghent, Belgium; ^4^Department of Internal Medicine and Pediatrics, Ghent University, Ghent, Belgium; ^5^Barriers in Inflammation, VIB Center for Inflammation Research, Ghent, Belgium; ^6^Department of Biomedical Molecular Biology, Ghent University, Ghent, Belgium; ^7^Laboratory of Cancer Immunoregulation, Immunology Program, Institute of Biomedical Sciences, Faculty of Medicine, University of Chile, Santiago, Chile; ^8^Laboratory of Immunoncology, Fundación Ciencia and Vida, Santiago, Chile; ^9^Facultad de Medicina y Ciencia, Universidad San Sebastián, Santiago, Chile; ^10^Division of Cell Medicine, Department of Life Science, Medical Research Institute, Kanazawa Medical University, Kahoku, Japan

**Keywords:** dendritic cells, immunity, IRE1, melanoma, unfolded protein response, XBP1, cDC1, antitumor immune response

## Abstract

In cancer, activation of the IRE1/XBP1s axis of the unfolded protein response (UPR) promotes immunosuppression and tumor growth, by acting in cancer cells and tumor infiltrating immune cells. However, the role of IRE1/XBP1s in dendritic cells (DCs) in tumors, particularly in conventional type 1 DCs (cDC1s) which are cellular targets in immunotherapy, has not been fully elucidated. Here, we studied the role of IRE1/XBP1s in subcutaneous B16/B78 melanoma and MC38 tumors by generating loss-of-function models of IRE1 and/or XBP1s in DCs or in cDC1s. Data show that concomitant deletion of the RNase domain of IRE1 and XBP1s in DCs and cDC1s does not influence the kinetics of B16/B78 and MC38 tumor growth or the effector profile of tumor infiltrating T cells. A modest effect is observed in mice bearing single deletion of XBP1s in DCs, which showed slight acceleration of melanoma tumor growth and dysfunctional T cell responses, however, this effect was not recapitulated in animals lacking XBP1 only in cDC1s. Thus, evidence presented here argues against a general pro-tumorigenic role of the IRE1/XBP1s pathway in tumor associated DC subsets.

## 1 Introduction

Dendritic cells (DCs) are major coordinators of antitumor immunity. A crucial arm of this response relies on effective activation of tumor specific cytotoxic CD8^+^ T cells endowed with the ability to eliminate cancer cells. Such activation depends on type 1 conventional dendritic cells (cDC1), which excel in cross-presentation of tumor-associated antigens (1–3), secrete immunostimulatory factors (2, 4–7), and sustain long-term CD8^+^T cell antitumor immunity (8). Additional DC subsets such as type 2 DCs (cDC2s), and a novel DC activation state termed ‘DC3’ can also boost antitumor CD4^+^ and CD8^+^ T cell responses (7, 9–11). In contrast, additional myeloid cells present in the tumor microenvironment (TME), such as tumor associated macrophages (TAM) and monocyte derived cells (MdC) display more complex pro or antitumorigenic roles that depend on the immunosuppressive environment (12). However, the molecular mechanisms that dictate antitumor roles in the different subsets of tumor associated DC/myeloid cells have not been fully elucidated.

An emerging intracellular pathway regulating myeloid cell biology is the inositol-requiring enzyme 1 alpha (IRE1) branch of the unfolded protein response (UPR), which is a cellular response maintaining the fidelity of the cellular proteome (13). Upon endoplasmic reticulum (ER) stress, the endoribonuclease (RNase) domain of IRE1 splices *Xbp1* mRNA, generating the transcription factor XBP1 spliced (XBP1s), master regulator of protein folding and ER biogenesis (13–15). The IRE1 RNase domain can also promote degradation of a subset of mRNAs/miRNAs in a process known as ‘regulated IRE1-dependent decay’ (RIDD) (16), which is a mechanism beginning to be understood in pathological settings including metabolism, inflammation and cancer (17–19).

Interestingly, IRE1 plays divergent roles in myeloid cell biology in steady state and tumor contexts. Steady state cDCs display constitutive IRE1 RNase activity without signs of canonical UPR activation (20). Among DC subsets, cDC1s (but not cDC2s) are markedly sensitive to perturbations in IRE1/XBP1s signaling, as genetic loss of the transcription factor XBP1 alters proteostatic programs and counter activates the RIDD branch (21). In turn, RIDD activation in cDC1s mediates the decay of various mRNAs involved in integrin expression, ER to golgi transport and antigen presentation, and on functional level, it regulates cell survival and antigen cross-presentation (21, 22). However, the role of IRE1 RNase in cDC1s in contexts extending beyond steady state are not fully understood.

In cancer, the IRE1/XBP1s branch can promote malignant tumor progression by acting directly on tumor cells (23, 24), T cells (25) and myeloid cells (26–28). For instance, DCs infiltrating ovarian cancer [typified by expression of the cDC2/MdC marker CD11b^+^ (29)] display persistent IRE1/XBP1s activation which blunts their immunostimulatory functions driving tumor progression (26). Similarly, in TAMs infiltrating melanoma models, IRE1/XBP1s activates expression of immunosuppressive genes that sustain tumor growth (27, 28). In fact, selective XBP1 deletion in DC/TAM delays melanoma tumor growth (26, 27). Whether the pro-tumorigenic role of the IRE1/XBP1s axis also operates to blunt cDC1-mediated antitumor responses has not been investigated. Thus, a correct delineation of the role of the enzyme in tumor cDCs is required to better understand the implications of therapeutic interventions targeting IRE1 in cancer.

Here, we sought to reveal the functional role of the IRE1/XBP1s axis in tumor cDCs in two immunoresponsive models: subcutaneous mouse B16/B78 melanoma and MC38 colon adenocarcinoma (2, 30) using a combination of single and double deficiency for IRE1 RNase and/or XBP1s driven by two conditional *knock-out* systems for deletion in the whole DC compartment or exclusively in cDC1s. Our data reveal that loss of the entire IRE1/XBP1s axis in DCs does not alter tumor growth or the effector profile of tumor infiltrating T cells. We observe that single deletion of XBP1s in the CD11c^+^ compartment results in modest acceleration of B16 melanoma growth accompanied by accumulation of exhausted CD8^+^T cells, which is associated with a compensatory increase in RIDD activity. Transcriptomic analysis revealed that the IRE1/XBP1s axis in tumor cDC1s controls a discrete set of proteostasis genes, without altering expression of immunosuppressive genes. Altogether, this work shows that the IRE1/XBP1s axis in tumor cDCs does not elicit a dominant pro-tumorigenic role in two tumor models known to respond to immunotherapeutic strategies.

## 2 Results

### 2.1 cDC1s constitutively activate IRE1 RNase in subcutaneous B16 and MC38 tumors

**Figure 1:**
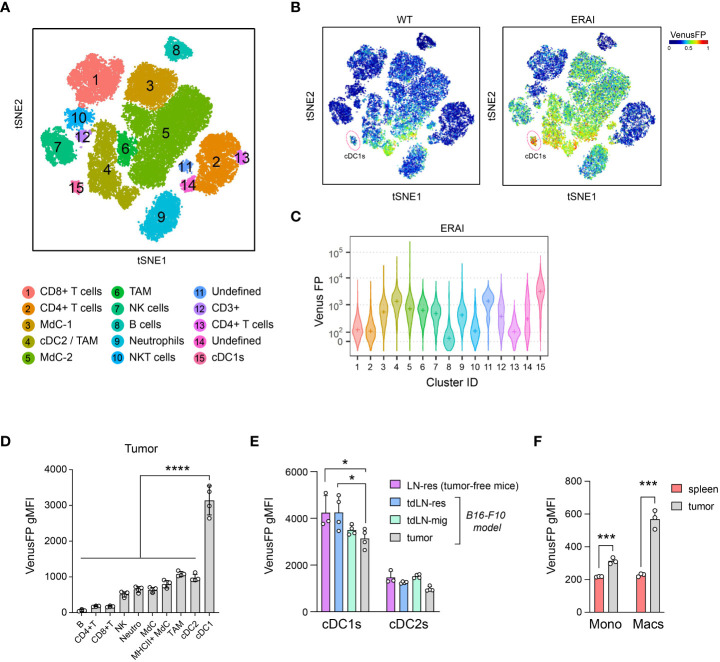
cDC1s constitutively activate IRE1 RNase in B16 tumors. B16-F10 melanoma cells were implanted intradermally on ERAI or control mice and 11 days after implantation, tumor tissue was analyzed by multicolor flow cytometry. n=4 mice per group, representative of two independent experiments. **(A-C)** t-SNE of 40.000 immune (CD45^+^) infiltrating cells from melanoma of ERAI or control mice. **(A)** Colors indicate unsupervised clustering by DBSCAN. See also **Supplementary Figures 1A, B**. **(B)** t-SNE map colored by VenusFP signal intensity from control (WT) or ERAI mice. cDC1 cluster is highlighted in a red circle. **(C)** VenusFP signal quantification across the different cell clusters identified in **(A)**. Median fluorescence intensity for VenusFP is depicted with a “+” inside each violin plot. See **Supplementary Figure 1C** for background signal in non-transgenic mice. **(D)** Quantification of VenusFP signal from manually gated immune populations from B16-F10-bearing ERAI mice (see gating strategy in **Supplementary Figure 2A**). gMFI, geometric Mean Fluorescence Intensity. ANOVA and Tukey post-test, **** p<0.0001. **(E)** Quantification of VenusFP signal from tumor and tumor draining lymph node migratory (mig) and resident (res) cDCs from tumor-bearing ERAI mice, or LN-resident cDCs from tumor-free ERAI mice (see gating strategy in **Supplementary Figure 2B**). * p<0.05, ANOVA and Tukey post-test. n=3 for tumor-free mice and n=4 for tumor-bearing mice, representative of two independent experiments, mean ± s.e.m. **(F)** Quantification of VenusFP signal from intratumoral and spleen monocytes (CD11b^hi^ Ly6C^hi^ cells), TAMs and splenic macrophages (F4/80^+^CD11c^-^). *** p<0.001, t-test. n= 3 mice, representative of two independent experiments, mean ± s.e.m.

**Figure 2:**
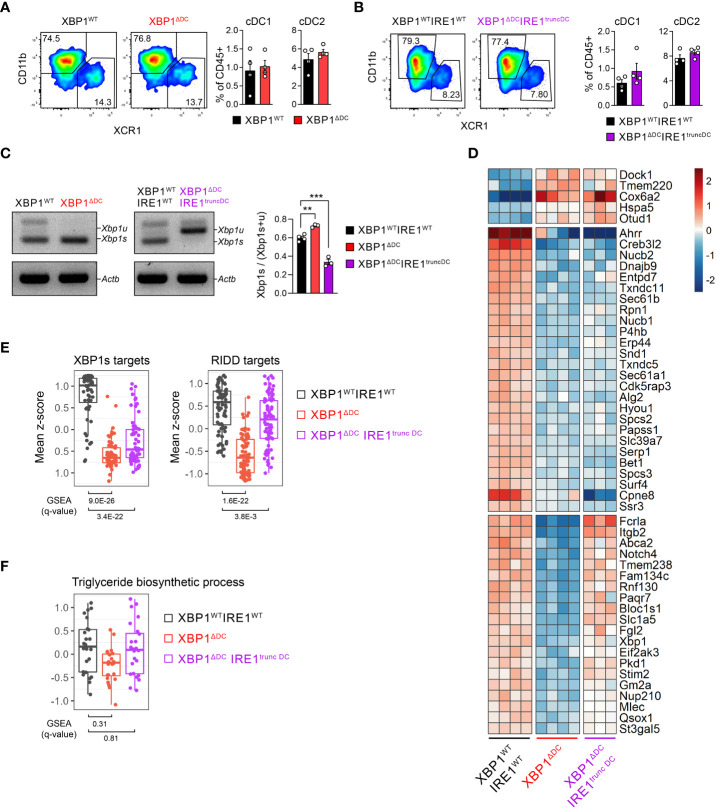
Transcriptomic signature associated with IRE1/XBP1s deficiency in tumor cDC1s. **(A, B)** Frequencies of intratumoral (B16-F10) cDC subsets from XBP1^ΔDC^ **(A)** or XBP1^ΔDC^ IRE1^truncDC^ **(B)** mice compared to control littermates. n=4 mice per group, representative of two independent experiments, mean ± s.e.m. **(C-F)** cDC1s were isolated by cell sorting from B16-F10 bearing XBP1^WT^IRE1^WT^, XBP1^ΔDC^ and XBP1^ΔDC^IRE1^truncDC^ mice. Control group includes 3 XBP1^fl/fl^ and 1 XBP1^fl/fl^IRE1^fl/fl^ cDC1 samples. **(C)** PCR analysis of *Xbp1* splicing. Xbp1u: Xbp1 unspliced; Xbp1s: Xbp1 spliced; Actb: beta actin. Bars depict quantification of bands by pixel density. ** p<0.01, ***p<0.001, two-tailed t-test. **(D)** Total RNA from tumor cDC1s was sequenced by RNA-seq. Heatmap of differentially expressed genes (DEGs) (adj p-value < 0.05 and |FC| > 1.5). **(E, F)** Comparison of mean z-scores for XBP1s- and RIDD-target genes from literature (34) **(E)** or genes of triglyceride biosynthetic process (GO:0019432) **(F)**. GSEA plots for these gene sets are shown in **Supplementary Figures 3C, D**. The q-values of GSEA are depicted below the box plots.

The TME contains activators of the IRE1/XBP1s axis that are detected by immune cells (25, 26, 31, 32). To identify relevant cell types activating IRE1 RNase in tumors, we analyzed the immune composition of B16 melanoma tumors of ERAI mice, a mouse strain that reports IRE1 RNase activity through expression of Venus Fluorescent Protein (VenusFP) fused with the sequence of *Xbp1s* (33) [validated in (21, 22, 31)]. Multi-color flow cytometry data (17-color) from ERAI tumors is depicted on a *t*-distributed stochastic neighbor embedding (*t*-SNE) map, identifying 15 cell clusters including CD8^+^ T cells, CD4^+^ T cells, monocyte-derived cells (MdCs), NK cells, NKT cells, B cells, neutrophils, cDC1s plus two undefined clusters (Cluster 11: CD4^+^ CD11c^+^ CD26^+^, Cluster 14: CD3^+^ CD4^+^ CD11b^int^ F4/80^+^ MHC-II^high^ CD11c^int^ CD26^high^) (**Figure 1A**, **Supplementary Figures 1A, B**). Analysis per cluster identified tumor cDC1s as the population with highest VenusFP fluorescence intensity (**Figures 1B, C**, **Supplementary Figure 1C**, cluster 15). cDC2s, MdCs, TAMs, neutrophils, NK cells and cells from cluster 11 also showed noticeable VenusFP levels, albeit lower than cDC1s; whereas CD4^+^ T cells, CD8^+^ T cells and B cells showed little or no VenusFP expression (**Figures 1B, C**). Manual gating analysis confirmed these findings (**Figure 1D**, **Supplementary Figure 1D**, see gating strategy in **Supplementary Figure 2A**), and similar results were observed in cDC1s infiltrating MC38 tumors (**Supplementary Figure 1E**). Data obtained with ERAI mice were further confirmed by PCR in cDC1s isolated from non-reporter animals bearing B16 tumors, which showed elevated *Xbp1* spliced/unspliced ratio (See XBP1^WT^, **Figure 2C**). Altogether, these data indicate that cDC1s display prominent activation of the IRE1 RNase/XBP1s axis in tumors.

We next interrogated whether the high IRE1 RNase activity observed in tumor cDC1s is a lineage-intrinsic signature or if it is a feature regulated by the tumor microenvironment. We compared IRE1 RNase activity in cDC1s from tumor, tumor draining lymph node (TdLN) and inguinal lymph nodes (LN) from tumor-free mice (gating strategy in **Supplementary Figure 2B**). VenusFP signal was elevated in all cDC1s analyzed but was not further increased in tumor cDC1s or in migratory cDC1s from TdLNs (**Figure 1E**). Conversely, a slight decrease in VenusFP signal was noted in tumor cDC1s when compared to TdLN-resident cDC1s from tumor-bearing or tumor-free mice (**Figure 1E**). Similar findings were observed in cDC1s from animals bearing MC38 tumors, suggesting that this may be a shared feature across tumor models (**Supplementary Figure 1F**). Of note, these observations were not replicated in monocytes or in macrophages from B16 tumors, which express higher VenusFP fluorescence in the tumor site compared to the spleen, indicating that the TME of melanoma is competent to elicit IRE1 activation in myeloid cells (**Figure 1F**), in line with previous findings (27). Thus, although tumor cDCs display elevated IRE1 RNase activity, this feature corresponds to a stable lineage-intrinsic trait not further enhanced by the TME.

### 2.2 Transcriptomic signature associated with IRE1/XBP1s deficiency in tumor cDC1s

To gain insights in the role of the IRE1/XBP1s axis in tumor DC biology, we generated double conditional *knock-out* mice lacking the IRE1 RNase domain and XBP1s in CD11c-expressing cells by crossing the *Itgax*-Cre mice line with *Xbp1*^fl/fl^ and *Ern1*^fl/fl^ floxed mice lines (35–37) (referred to as “XBP1^ΔDC^IRE1^truncDC^ mice”); and single conditional *knock-out* mice (referred to as “XBP1^ΔDC^ mice”) lacking XBP1s in CD11c-expressing cells (see methods for details). The rationale for studying single deletion of XBP1s in DCs is because IRE1 can promote multiple effects via its RNase domain, independent of the XBP1s transcriptional activity and involving degradation of mRNAs/miRNAs through the RIDD branch (16–19), which is observed in steady state cDC1s (21, 22). Also, the XBP1^ΔDC^ mice line has been previously studied in other tumor models, showing delay in ovarian cancer progression (26). XBP1^ΔDC^ and XBP1^ΔDC^IRE1^truncDC^ mice are compared to control animals (*Xbp1*^fl/fl^ and *Xbp1*^fl/fl^*Ern1*^fl/fl^ littermates with no expression of Cre, respectively). The frequency of tumor cDCs was unaltered in XBP1^ΔDC^ and XBP1^ΔDC^IRE1^truncDC^ mice compared to control littermates upon implantation of B16F10 tumor cells (**Figures 2A, B**). We measured IRE1 RNase activity in tumor cDC1s from XBP1^ΔDC^ and XBP1^ΔDC^IRE1^truncDC^ mice by analyzing the expression of *Xbp1* spliced and unspliced mRNA. Although DCs from XBP1^ΔDC^ mice are unable to synthesize XBP1s protein, these cells still generate mRNA containing the sites for cleavage by IRE1, allowing assessment of IRE1 RNase activity by PCR (Scheme in **Supplementary Figure 3A**). Whereas tumor cDC1s from control mice displayed high levels of *Xbp1* spliced mRNA (**Figure 2C**), tumor cDC1s isolated from XBP1^ΔDC^IRE1^truncDC^ mice were unable to induce *Xbp1* splicing due to the lack of a functional IRE1 RNase domain (**Figure 2C**). In contrast, tumor cDC1s from XBP1^ΔDC^ mice showed signs of IRE1 hyperactivation, as indicated by higher expression of the *Xbp1* spliced over the unspliced form (**Figure 2C**).

Considering that XBP1s coordinates expression of immunosuppressive genes in TAMs in melanoma (27, 28), we determined the transcriptomic signature of cDC1s isolated from B16 melanoma tumors of control, XBP1^ΔDC^ or XBP1^ΔDC^IRE1^truncDC^ mice by bulk RNA sequencing (RNA-seq). Surprisingly, only 51 differentially expressed genes (DEG) were identified among XBP1^ΔDC^ or XBP1^ΔDC^IRE1^truncDC^ tumor cDC1s (**Figure 2D**, **Supplementary Table 1**), corresponding mainly to ER proteins and constituents of the response to misfolded proteins (**Supplementary Figure 3B**). Gene Set Enrichment Analysis (GSEA) confirmed that canonical XBP1-target genes (34) were downregulated in both XBP1s-deficient and IRE1/XBP1-deficient tumor cDC1s (**Figure 2E**, **Supplementary Figure 3C**). These include genes coding for protein disulfide isomerases, chaperones, glycosylation proteins, proteins involved in transport to the ER and from the ER to Golgi. Furthermore, XBP1 single deficient but not IRE1 RNase/XBP1 double deficient cDC1s showed also a decrease in the levels of the RIDD-targets (34) (**Figure 2E**, **Supplementary Figure 3C**) *Bloc1s1*, *St3gal5*, *Itgb2*, *Rpn1, Erp44, Mlec, Rnf130, Abca2, Stim2, Gm2a, Tmem238, and Paqr7* (16, 21, 34). These data suggest that loss of XBP1s elicits RIDD in tumor cDC1s.

Notably, expression of XBP1s-driven triglyceride biosynthesis genes reported to curtail the function of MoDC/cDC2 in ovarian cancer models (26) was unaltered in XBP1^ΔDC^ or XBP1^ΔDC^IRE1^truncDC^ mice tumor cDC1s, (**Figure 2F**, **Supplementary Figure 3D**). These results demonstrate that cDC1s isolated from B16 tumors maintain a stable transcriptomic signature and that deletion of the entire IRE1/XBP1s axis in tumor cDC1s only alters a discrete subset of genes related with protein homeostasis without affecting expression of pro-tumorigenic genes.

### 2.3 Double deficiency of IRE1/XBP1s in DCs does not regulate melanoma tumor growth

**Figure 3:**
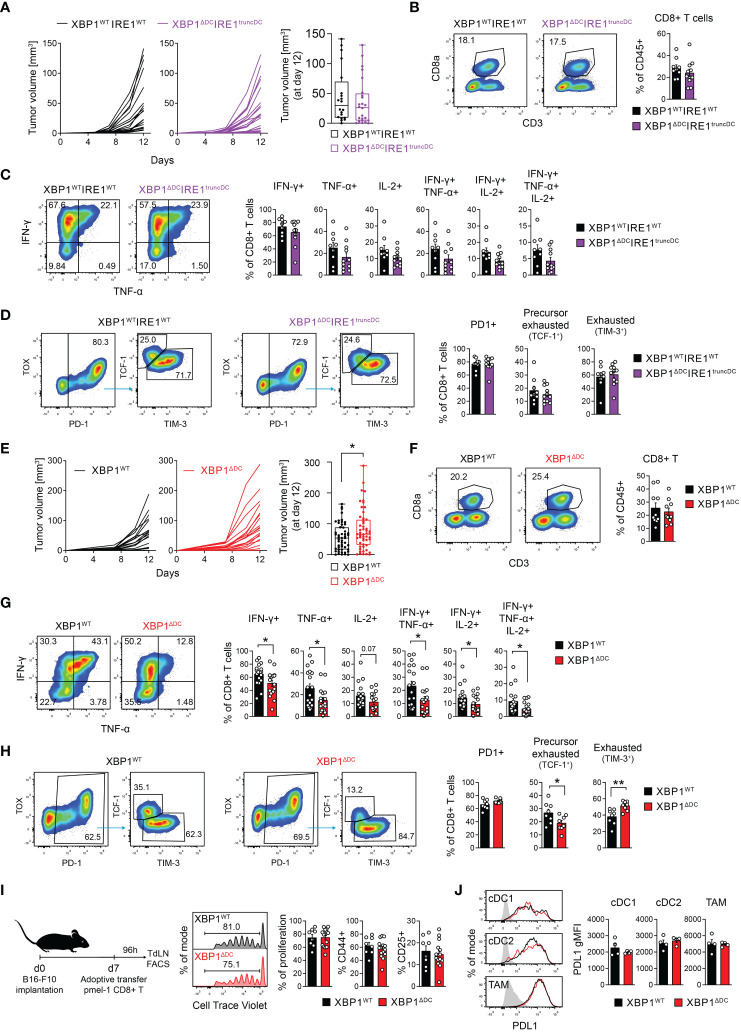
XBP1s deficiency in DCs elicits modest acceleration of tumor growth and reduced frequencies of effector/precursor exhausted T cells. **(A–D)** XBP1^WT^IRE1^WT^ and XBP1^ΔDC^IRE1^truncDC^ mice were implanted with B78ChOVA cells. **(A)** Tumor growth curves monitored over a period of 12 days. Boxplot of tumor size at day 12 post implantation. n=24 mice (XBP1^WT^IRE1^WT^) or 28 mice (XBP1^ΔDC^IRE1^truncDC^), data pooled from 5 independent experiments. **(B)** Frequencies of intratumoral CD8^+^ T cells. **(C)** Frequencies of cytokine producing intratumoral CD8^+^ T cells after *ex vivo* stimulation with PMA/Ionomycin in the presence of BFA. **(D)** Precursor exhausted (PD1^+^ TCF1^+^ TIM3^neg^) and exhausted (PD1^+^ TCF1^neg^ TIM3^+^) CD8^+^ T cell frequencies in tumors. Gated on CD3^+^CD8^+^ T cells. **(B–D)** n=9 mice (XBP1^WT^IRE1^WT^) or 11 mice (XBP1^ΔDC^IRE1^truncDC^), data pooled from three independent experiments, mean ± s.e.m. **(E–H)** XBP1^WT^ and XBP1^ΔDC^ mice were implanted with B78ChOVA cells. **(E)** Tumor growth curves monitored over a period of 12 days. n=19 mice per group, pooled from 4 independent experiments. Boxplot of tumor size at day 12 post implantation. * p<0.05, two-tailed t-test. n= 51 mice (XBP1^WT^) or 53 mice (XBP1^ΔDC^) from animals used throughout this study, pooled data from 12 independent experiments. **(F)** Frequencies of intratumoral CD8^+^ T cells. n=10 mice per group, pooled data from three independent experiments, mean ± s.e.m. **(G)** Frequencies of cytokine producing tumor CD8^+^ T cells after *ex vivo* cursive stimulation with PMA/Ionomycin in the presence of BFA. *p < 0.05, two-tailed Mann-Whitney test. n=18 mice (XBP1^WT^) or 17 mice (XBP1^ΔDC^), pooled data from 4 independent experiments, mean ± s.e.m. **(H)** Precursor exhausted (PD1^+^ TCF1^+^ TIM3^neg^) and exhausted (PD1^+^ TCF1^neg^ TIM3^+^) CD8^+^ T cell frequencies in tumors. Gated on CD3^+^CD8^+^ T cells. * p<0.05, **p<0.01, two-tailed t-test. n=8 mice per group, pooled data from two independent experiments, mean ± s.e.m. **(I)** Melanoma specific (pmel-1) CD8^+^ naïve T cells were adoptively transferred into B16-F10-bearing XBP1^WT^ and XBP1^ΔDC^ at day 7 after tumor implantation. Four days later, proliferation and CD44/CD25 expression of transferred cells in TdLN was quantified by FACS. n=8 mice (XBP1^WT^) or 15 mice (XBP1^ΔDC^), data pooled from two independent experiments, mean ± s.e.m. **(J)** PD-L1 expression in tumor cDC1, cDC2 and TAM from B78ChOVA-bearing XBP1^WT^ and XBP1^ΔDC^ mice. n=4 mice per group, representative data from two independent experiments.

To evaluate if the loss of the IRE1/XBP1s axis in DCs alters the course of tumor growth, we studied immunogenic B78/B16 lines expressing the model antigen ovalbumin. We studied the immunogenic B78ChOVA subcutaneous tumor model that is a B16 variant expressing OVA and mCherry fluorescent protein (2) and that elicits cDC1-mediated antitumor CD8^+^ T cell responses (2, 3). After subcutaneous B78ChOVA implantation, tumor growth in XBP1^ΔDC^IRE1^truncDC^ animals was comparable to that observed in control animals (**Figure 3A**). Also, similar frequencies of tumor infiltrating CD8^+^ T cells were found in XBP1^ΔDC^IRE1^truncDC^ and control animals (**Figure 3B**) and no differences were observed in IFN-γ-producing CD8^+^T cells, TNF-producing CD8^+^T cells, IL-2-producing CD8^+^T cells between tumors of XBP1^ΔDC^IRE1^truncDC^ mice and control animals (**Figure 3C**). Similar results were observed for CD4^+^ T cells (**Supplementary Figures 4A, B**). We extended our analysis to measure parameters of CD8^+^ T cell exhaustion (38–40) and found similar proportions of ‘exhausted’ TIM-3^+^CD8^+^ T cells and ‘precursor exhausted’ TCF-1^+^CD8^+^ T cells in tumors from XBP1^ΔDC^IRE1^truncDC^ mice compared to controls (**Figure 3D**). These data show that deletion of the IRE1 RNase/XBP1s branch of the UPR in DCs does not alter the course of melanoma tumor growth or the generation of effector/exhausted T cells at the tumor site.

### 2.4 XBP1s deficiency in DCs elicits modest acceleration of tumor growth and reduced frequencies of effector/precursor exhausted T cells

We investigated if XBP1^ΔDC^ mice display alterations in tumor growth. Animals bearing single deficiency of XBP1s in DCs showed a modest acceleration of B78ChOVA growth and larger tumor size than tumors from XBP1^WT^ mice on day 12 post implantation (**Figure 3E**). Tumor growth kinetics of subcutaneous MC38 tumors from XBP1^ΔDC^ and XBP1^WT^ mice did not reach statistical significance (**Supplementary Figure 5A**).

XBP1^WT^ and XBP1^ΔDC^ mice show comparable B78ChOVA tumor immune cell composition and T cell infiltration (**Figure 3F** **Supplementary Figure 6A, B**). However, B78ChOVA tumors from XBP1^ΔDC^ mice contained lower frequencies of IFN-γ-producing and TNF-producing CD8^+^ T cells, decreased frequencies of IFN-γ^+^TNF^+^ CD8^+^ T cells and IFN-γ^+^TNF^+^IL-2^+^ CD8^+^ T cells (**Figures 3F, G**). Some of these observations were also extended to CD4^+^ T cells, which showed decreased frequencies of IFN-γ^+^ and IFN-γ^+^TNF^+^ CD4^+^ T cells in B78ChOVA tumors and in MC38 tumors (**Supplementary Figure 6C**, **Supplementary Figure 5B, C**). B78ChOVA tumors from XBP1^ΔDC^ mice also show reduced percentages of precursor exhausted TCF-1^+^CD8^+^ T cells compared to control animals (**Figure 3H**, **Supplementary Figure 6D**).

Interestingly, the reduced frequencies of effector T cells in B78ChOVA tumors from XBP1^ΔDC^ mice were not associated with defective cross-presentation of a model antigen in melanoma, as XBP1^WT^ and XBP1^ΔDC^ mice lines contained similar frequencies of endogenous OVA-specific CD8^+^ T cells in TdLN and tumors (**Supplementary Figure 6E, F**). A similar response was obtained when tracking proliferation/early activation of CD8^+^ T cells isolated from pmel mice, which possess transgenic CD8^+^ T cells bearing a TCR selective for the melanoma-associated antigen gp100 (41) (**Figure 3I**). Also, cDC1, cDC2 and TAMs from melanoma tumors of XBP1^ΔDC^ mice expressed normal levels of PD-L1 (**Figure 3J**), an immunoregulatory molecule target of checkpoint blockade therapy which expression is attributed to the IRE1/XBP1s axis in tumor macrophages (28). In addition, *in vitro* generated bone marrow cDC1s from XBP1^WT^ and XBP1^ΔDC^ mice were equally able to produce IL-12 upon stimulation with B78ChOVA lysates (**Supplementary Figure 7**). We conclude that XBP1 deletion in tumor-associated DCs tunes effector T cell responses independent of cross-presentation or canonical DC activation. Altogether, these data show that double ablation of the IRE1/XBP1 axis in DCs does not alter the course of tumor growth, whereas single XBP1 deficiency in DCs results in mild changes in tumor growth and T cell effector/exhausted profiles at the tumor site. The discrepancies between double versus single *knock-out* models may be attributed to RIDD counteractivation in XBP1-deficient tumor cDC1s (**Figures 2D, E**), which is a phenotype reported in XBP1-*knock-out* cDC1s in steady state (21, 22). In fact, tumor cDC1s, cDC2s and TAMs from XBP1^ΔDC^ mice display signs of RIDD at protein level, as surface expression of the integrin CD11c, an obligate dimeric partner of the RIDD substrate *Itgb2* (coding the integrin CD18) (21) is reduced in tumor cDC1s from XBP1^ΔDC^ mice (**Supplementary Figure 6G**).

### 2.5 cDC1-specific loss of the IRE1/XBP1 signaling axis does not alter B16/MC38 tumor growth or the T cell compartment

Finally, we interrogated if selective IRE1/XBP1s deletion in the cDC1 compartment could alter the course of tumor growth and T cell responses. To specifically target cDC1 compartment for genetic deletion of IRE1 RNase and/or XBP1s, we crossed the *Xcr1*-Cre transgenic mice line (42) with Ern1^fl/fl^ x XBP1^fl/fl^ mice (referred to as ‘XBP1^ΔcDC1^IRE1^trunc-cDC1^ mice’), or with XBP1^fl/fl^ mice (‘XBP1^ΔcDC1^ mice’). XBP1^ΔcDC1^IRE1^trunc-cDC1^ and XBP1^ΔcDC1^ mice showed normal tumor cDC infiltration in MC38 (**Supplementary Figures 8A, B**) and tumor cDC1s efficiently recombined loxP-flanked sites (**Supplementary Figure 8C, D**).

**Figure 4:**
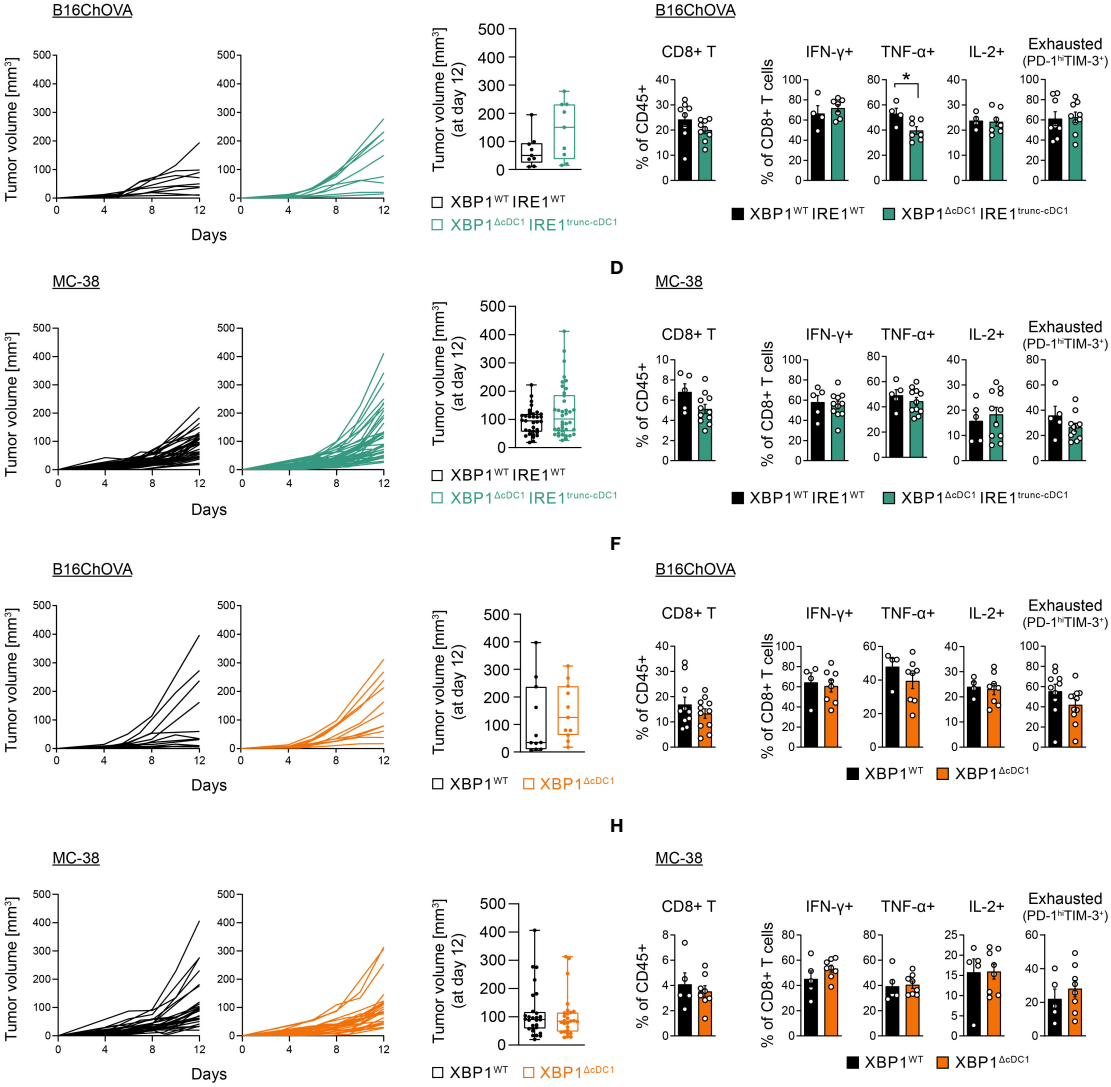
cDC1-specific loss of the IRE1/XBP1 signaling axis does not alter B16/MC38 tumor growth or T cell compartment. **(A–D)** XBP1^WT^IRE1^WT^ and XBP1^ΔcDC1^IRE1^trunc-cDC1^ mice were implanted with B16ChOVA cells **(A, B)** or MC38 cells **(C, D)**. **(A)** Tumor growth curves monitored over a period of 12 days. Boxplot of tumor volume at day 12 post implantation. n=9 mice per group, data pooled from 2 independent experiments. **(B)** CD8^+^ T cell frequencies, cytokine production and PD-1^hi^ TIM-3^+^ frequencies. n=4-8 mice (XBP1^WT^IRE1^WT^) or n=7-9 mice (XBP1^ΔcDC1^IRE1^trunc-cDC1^), data pooled from two independent experiments, mean ± s.e.m. * p<0.05, two-tailed t test. **(C)** Tumor growth curves monitored over a period of 12 days. Boxplot of tumor volume at day 12 post implantation. n=33 mice (XBP1^WT^IRE1^WT^) or 38 mice (XBP1^ΔcDC1^IRE1^trunc-cDC1^), data pooled from 9 independent experiments. **(D)** CD8^+^ T cell frequencies, cytokine production and PD-1^hi^ TIM-3^+^ frequencies. n=5 mice (XBP1^WT^IRE1^WT^) or n=11 mice (XBP1^ΔcDC1^IRE1^trunc-cDC1^), data pooled from two independent experiments, mean ± s.e.m. **(E–H)** XBP1^WT^ and XBP1^ΔcDC1^ mice were implanted with B16ChOVA cells **(E, F)** or MC38 cells **(G, H)**. **(E)** B16ChOVA growth curves monitored over a period of 12 days. Boxplot of tumor volume at day 12 post implantation. n=11 mice (XBP1^WT^) and n=11 mice (XBP1^ΔcDC1^), data pooled from 2 independent experiments. **(F)** CD8^+^ T cell frequencies, cytokine production and PD-1^hi^ TIM-3^+^ frequencies. n=4-10 mice (XBP1^WT^) or n=8-11 mice (XBP1^ΔcDC1^), data pooled from two independent experiments, mean ± s.e.m. **(G)** Tumor growth curves monitored over a period of 12 days. Boxplot of tumor volume at day 12 post implantation. n=28 mice (XBP1^WT^) and n=27 mice (XBP1^ΔcDC1^), data pooled from 8 independent experiments. **(H)** CD8^+^ T cell frequencies, cytokine production and PD-1^hi^ TIM-3^+^ frequencies. n=5 mice (XBP1^WT^) or n=8 mice (XBP1^ΔcDC1^), data pooled from two independent experiments, mean ± s.e.m.

We next carried out tumor kinetic studies. Analysis revealed that XBP1^ΔcDC1^IRE1^trunc-cDC1^ mice showed normal B16ChOVA melanoma tumor growth (**Figure 4A**), and normal CD8^+^/CD4^+^ T cell tumor infiltration and function compared to control littermates, and a modest decrease in TNF^+^ CD8^+^ T cells (**Figure 4B**, **Supplementary Figure 8E**). Similar results were obtained in MC38 tumor models (**Figures 4C, D**, **Supplementary Figure 8E**). In agreement with previous results, these data indicate that loss of IRE1 RNase/XBP1s in cDC1s does not impact the outcome of tumor growth or antitumor T cell function.

In addition, analysis of mice carrying single deletion of XBP1s in cDC1s (XBP1^ΔcDC1^) also showed comparable B16ChOVA tumor size with control counterparts (**Figure 4E**), along with normal effector/exhausted T cell responses (**Figure 4F**, **Supplementary Figure 8F**). Similar results were also observed in MC38 tumors (**Figures 4G, H**, **Supplementary Figure 8F**). As seen with XBP1^ΔDC^ mice, tumor cDC1s from XBP1^ΔcDC1^ mice also showed signs of RIDD, as indicated by an increase in XBP1s splicing ratio (**Supplementary Figure 8G**) and loss of CD11c surface expression (**Supplementary Figure 8H**), albeit to a lower extent than observed with *Itgax*-Cre mice line (**Supplementary Figure 6G**). Altogether, these data suggest that alterations in the IRE1/XBP1s axis in the tumor cDC1 compartment are not sufficient to shift the balance of antitumor T cell responses.

## 3 Discussion

The IRE1/XBP1s axis is a critical regulator of immunity and cancer (32, 43, 44). The differential mechanisms by which IRE1 signaling integrates the intensity and duration of ER stress to regulate cell fate is particularly noticed in the immune system, with cells such as cDC1s, B cells or NK cells that opt for an intact IRE1/XBP1s axis to maintain cellular health (22, 31, 45–47), and cells including TAM/MdCs or intratumoral T cells, which acquire dysfunctional phenotypes upon enforced IRE1/XBP1s activation at the tumor site (25, 28, 32).

Here, we report a broad approach for studying the role of the IRE1/XBP1s axis in tumor DCs/cDC1s, by using a combination of two different immunoresponsive tumor models, two different conditional *Cre* lines for selective deletion in the DC or cDC1 compartment respectively, plus single and double deletion of the IRE1/XBP1s axis. Our results indicate that full deletion of the IRE1 RNase/XBP1s branch of the UPR in the whole DC compartment or selectively in cDC1s does not influence the course of B16/B78 or MC38 tumor growth. This result is unexpected, given the reported pro-tumorigenic roles of the pathway in tumor myeloid cells including TAMs and MoDC/cDC2 (26–28). In fact, an aspect uncovered in this study is that tumor cDC1s display stable IRE1 RNase activity, which is not further induced by the TME. Furthermore, IRE1/XBP1 ablation in tumor cDC1s controls a discrete set of genes related to proteostatic programs without altering pro-tumorigenic programs seen in other tumor myeloid cell subsets (26–28). The question as to why cDC1s remain refractory to the detrimental effects of IRE1/XBP1s activation in tumors remains to be investigated. Interestingly, in contrast to tumor cDC1s, we corroborate that tumor monocytes and macrophages activate IRE1 RNase at the B16 tumor site, indicating that tumor-infiltrating myeloid cells set different thresholds for triggering IRE1/XBP1s activity. Further work is required to reveal how these different modes of IRE1 RNase activation are wired to functional outputs in tumor myeloid cells.

Our work also uncovers differences between double IRE1 RNase/XBP1s deletion and single XBP1s deletion in DCs, as the latter but not the former mice line exhibit increased melanoma tumor growth, reduced effector cytokine-producing T cells and reduced precursor exhausted T cells at the tumor site. These IRE1 RNase-dependent effects are likely attributed to compensatory RIDD activation, which is reported to occur upon genetic loss of XBP1s in a subset of cells including cDC1s, plasma cells and hepatocytes (21, 34, 48, 49). In this context, the activation of RIDD upon XBP1 loss is a matter that should be carefully assessed when studying XBP1 deficient models. However, the intensity of RIDD elicited in response to XBP1s deletion in tumor cDC1s is arguably non-physiological and it may not be recapitulated during tumor growth in wild type conditions. Thus far, the role of RIDD in physiology remain speculative and future studies should investigate if certain tumors can cause RIDD to fine tune the function of tumor cDC1s. The transcriptomic analysis provided here does not identify potential RIDD targets with direct immunoregulatory functions, suggesting that additional mechanisms (such as microRNA control, metabolic regulation) may be responsible for the phenotypes observed. Also, XBP1^ΔDC^ mice have normal cross-presentation of tumor antigens, which is distinct to observations in steady state cDC1s from the same mouse line (21). A possibility accounting for these differences is that tumors display additional antigen presentation strategies not commonly observed in steady state, such as crossdressing (50, 51), which may bypass the effect of XBP1s loss. Also, *tapbp* mRNA, a reported RIDD target in splenic cDC1s (21) is not found as DEG in XBP1 deficient tumor cDC1s. In fact, one candidate identified in this analysis as a potential RIDD substrate is the ER-resident FC receptor Like A (*Fcrla)*, which has been previously identified as part of a BATF3/IRF8 transcriptional program that confers tumor immunogenicity in cDC1s independently of cross-presentation (52). Thus, whether RIDD -dependent degradation of *Fcrla* mRNA by cDC1s contribute to increase tumor growth is a hypothesis that remains to be formally demonstrated. In addition, and as reported in infection (53), tumors may potentially induce ‘emergency DC-poiesis’, a process in which tissues receive a large influx of pre-DCs from the bone marrow that rapidly differentiate into cDCs to supply demand. Whether this type of process occurs in tumors and if newly differentiated DCs show differential responses to ablation of UPR components remains to be investigated.

Our data also show that the mechanisms controlled by IRE1/XBP1s in other tumor myeloid cells cannot be extrapolated to intratumoral cDCs. PD-L1 expression, a reported IRE1 target in TAMs (28), is not regulated by the pathway in tumor cDCs. Also, the core of triglyceride biosynthesis genes regulated by XBP1s in DCs from ovarian cancer (26) are not found as DEG in XBP1 deficient tumor cDC1s from this study. These differences may explain the divergent outcomes in tumor growth showed by the same XBP1^ΔDC^ mice line in the B78ChOVA model compared to ovarian cancer (26) or with mice lacking XBP1s in macrophages in melanoma studies (27). Combining this data, we must consider that the IRE1 outputs in tumor DCs may drastically differ depending on the subset and the cancer type.

Finally, multiple efforts are focused on the development of pharmacological compounds targeting the IRE1 RNase active site and XBP1s *in vivo*, many of which have shown translational potential in cancer (54–57). A study revealed that RIDD regulates expression of the MHC-I heavy chain mRNAs in DCs and that pharmacological IRE1 RNase inhibition attenuates tumor growth in 4T1 and CT26 models, by a mechanism proposed to be dependent on DC cross-presentation (55). Even though we do not find MHC-I heavy chain mRNAs as DEGs in this study, and we do not find an improved antitumor response in XBP1^ΔDC^IRE1^truncDC^ animals, future studies are required to comprehensively integrate these findings. Based on the work presented here, our data argues against a general pro-tumorigenic role for IRE1/XBP1s axis in cDCs from immunoresponsive B16/B78 and MC38 tumors.

## 4 Materials and methods

### 4.1 Experimental model and subject details

#### 4.1.1 Mice

ER-stress Activation Indicator (ERAI) (33) and non-transgenic control (WT) littermates, XBP1^WT^[XBP1fl/fl (35)], XBP1^ΔDC^ [XBP1fl/fl x *Itgax*-Cre (36)], XBP1^WT^IRE1^WT^ [XBP1fl/fl x IRE1fl/fl (37)], XBP1^ΔDC^IRE1^truncDC^ (XBP1fl/fl x IRE1fl/fl x *Itgax* -Cre), XBP1^ΔcDC1^ [XBP1fl/fl x *Xcr1*-Cre (42)] and XBP1^ΔcDC1^IRE1^trunc-cDC1^ (XBP1fl/fl x IRE1fl/fl x *Xcr1*-Cre) were bred at the animal facilities of Universidad de Chile, Fundación Ciencia & Vida or the VIB-UGent institute, under specific pathogen-free conditions. Briefly, XBP1fl/fl mice allow cre-mediated recombination of the exon 2 of *Xbp1*, resulting in absence of the transcription factor (21), while IRE1fl/fl mice delete exons 20-21 of the *Ern1* gene upon cre-mediated recombination, which generates a truncated IRE1 isoform lacking the RNase domain (21, 37). Pmel-1 mice (41) were kindly donated by Dr F. Salazar-Onfray. All mice were kept on a C57BL/6 background. Litters with mice of both sexes at 6–14 weeks of age were used for experiments.

#### 4.1.2 Cell lines

B78ChOVA cells were kindly provided by Dr. Matthew Krummel (UCSF) (2). B16-F10 cells were obtained from ATCG (#CRL-6475). MC38 cell line (58) was provided by Dr. Álvaro Lladser (FCV) (Universidad San Sebastian) and by Prof. Dr. Jannie Borst (LUMC). OP9 cells expressing Notch ligand DL1 (OP9-DL1) (59) were kindly provided by Dr. Juan Carlos Zuñiga-Pflucker (Sunnybrook Research Institute, Canada). B16-F10-mCherry-OVA originate from (60). Cells were cultured under standard conditions prior to injection into mice. Briefly, cells were cultured in DMEM (B78ChOVA/B16ChOVA/MC38) or RPMI-1640 (B16-F10/MC38) supplemented with 10% v/v inactivated fetal bovine serum (FBS, Gibco), 100 U/mL penicillin (Corning), 100 µg/mL streptomycin (Corning) and 0.55 mM 2-Mercaptoethanol (Gibco). For MC38 culture, media was supplemented additionally with non-essential amino acids (ThermoFisher Scientific), 1 mM sodium pyruvate (ThermoFisher Scientific) and 10 mM HEPES (Gibco). Cells were cultured on T75 tissue-culture treated plastic flasks at 37°C, 5% CO_2_. Cells were split every other day. OP-DL1 cells were cultured in MEM-alpha medium supplemented with 20% FBS (Gibco), 100 U/mL penicillin (Corning), 100 µg/mL streptomycin (Corning), 1mM sodium pyruvate (Gibco) and 0.55 mM 2-Mercaptoethanol (Gibco).

### 4.2 Method details

#### 4.2.1 Tumor model

Tumor cell lines were harvested, washed with PBS, and resuspended in a final injection volume of 50 μl PBS. 5x10^5^ (B16-F10/B78ChOVA/B16ChOVA) or 1x10^6^ (MC38) tumor cells were injected in the right flank of shaved mice intradermally and allowed to grow for 10-15 days. For tumor growth curves, tumor size was determined by two orthogonal measurements with a caliper and the volume was estimated as (width^2 x length)/2.

#### 4.2.2 Preparation of cell suspensions

Tumors were minced and digested in HBSS with Collagenase D (1 mg/mL, Roche) or Collagenase A (2mg/ml, Roche) and DNAse I (50 μg/mL, Roche) for 30 minutes at 37°C in a water bath. Digested tissue was then passed through a 70 μm cell strainer, followed by red blood cell lysis with RBC lysis buffer (Biolegend). Single cells were kept on ice.

For whole intratumoral immune cell profiling and DC stainings, CD45-biotin magnetic positive selection (MACS, Miltenyi) was performed to enrich for total tumor immune infiltrate.

For intratumoral T cell stainings, hematopoietic cells were enriched by density gradient centrifugation with 40/70 Percoll (GE Healthcare) for 20 min at 700xg.

Tumor draining lymph nodes (tdLNs) and spleens were minced and digested in RPMI 1640 with Collagenase D (1 mg/mL, Roche) and DNAse I (50 μg/mL, Roche) for 45 minutes at 37°C in a water bath. Digested tissue was then passed through a 70 μm cell strainer and single cells were kept on ice. For spleens, red blood cells were lysed using RBC lysis buffer (Biolegend).

#### 4.2.3 Bone marrow derived cDC1s generation and tumor lysate stimulation

Bone marrow cells from femurs and tibias were cultured in presence of 100 ng/ml recombinant human FLT3-L (Peprotech). After three days of differentiation, cells were plated onto a monolayer of OP9-DL1 stromal cells and co-cultured for additional 6 days in P24 plates as previously reported (61, 62).

For tumor lysate preparation B78ChOVA cells were washed twice with PBS, resuspended at 8x10^6^ cells/mL in RPMI supplemented with 10% FBS and aliquoted in cryotubes. Cell suspensions were subjected to heat-shock (42°C for 60 min) followed by three cycles of freeze/thaw (liquid nitrogen/waterbath at 37°C). Tumor lysates were stored at -80°C until use.

BM-derived cDC1s were harvested and plated with B78ChOVA lysates (50 uL/mL) in round-bottom p96 plates. After 14h, Brefeldin A (GolgiPlug, BD) was added and four hours later, cells were harvested. Next, cells were fixed and permeabilized using BD Cytofix/Cytoperm fixation/permeabilization kit (BD) followed by intracellular staining of IL-12p40 and analysis by FACS.

#### 4.2.4 RNA isolation, Xbp1s splicing assay and RT-qPCR

RNA was isolated by using the RNeasy Plus Micro Kit (Qiagen) according to manufacturer’s protocol. Amplified cDNA was prepared by using the Ovation PicoSL WTA System V2 kit (TECAN) and cleaned up with the MinElute Reaction Cleanup kit (Qiagen). Conventional PCR was performed with GoTaq G2 Green Master Mix 2X (Promega) on a thermal cycler (BioRad). The following primers were used for conventional PCR amplification of total Xbp1: Fwd: 5’-ACACGCTTGGGAATGGACAC-3’ and Rev: 5’-CCATGGGAAGATGTTCTGGG-3’ (21); and for beta actin (*Actb*): Fwd 5’-GTGACGTTGACATCCGTAAAGA-3’ and Rev: 5’-GCCGGACTCATCGTACTCC-3’. PCR products were analyzed on agarose gels. RT-qPCR was performed with the SensiFAST SYBR No-ROX kit (Bioline) on a LightCycler 480 (Roche). mRNA expression was analyzed using qbase+ 3.2 (Biogazelle). The following primers were used for RT-qPCR: Ern1 (exon19-20): Fwd: 5’-TGCTGAAACACCCCTTCTTC-3’ and Rev: 5’-GCCTCCTTTTCTATTCGGTCA-3’. Xbp1 (exon2): Fwd: 5’-CAGCAAGTGGGGATTTGG-3’ and Rev: 5’-CGTGAGTTTTCTCCCGTAAAAG-3’. Ywhaz: Fwd: 5’-CTCTTGGCAGCTAATGGGCTT-3’ and Rev: 5’-GGAGGTGGCTGAGGATGGA-3’. Sdha: Fwd: 5’- TTTCAGAGACGGCCATGATCT -3’ and Rev: 5’-TGGGAATCCCACCCATGTT-3’.

#### 4.2.5 Flow cytometry and cell sorting

For surface staining, cells were incubated with anti-Fc receptor antibody and then stained with fluorochrome-conjugated antibodies (Supplementary Reagents and Tools Table) in FACS buffer (PBS + 1% FBS + 2mM EDTA) for 30 min at 4°C. Viability was assessed by staining with fixable viability Zombie (BioLegend), Fixable Viability Dye (eBioscience) or LIVE/DEAD fixable (Invitrogen). Flow cytometry was performed on BD LSR Fortessa or FACSymphony (BD Biosciences) instruments using FACSDiva software (BD Biosciences). Analysis of flow cytometry data was done using FlowJo software. Cell sorting was performed using FACS Aria II and Aria III, and FACS Symphony S6 (BD Biosciences).

#### 4.2.6 Transcription factors and granzyme B intracellular staining

After surface staining, cells were fixed and permeabilized using Foxp3 transcription factor staining set (eBioscience) followed by intracellular staining of transcription factors (Foxp3, Tcf1, Tox) and/or granzyme B as indicated by the manufacturer protocol.

#### 4.2.7 T cell stimulation and intracellular cytokine staining

Tumor and TdLN cell suspensions were stimulated *ex-vivo* with 0.25 μM phorbol 12- myristate 13-acetate (PMA; Sigma) and 1 μg/mL Ionomycin (Sigma) at 37°C and 5% CO_2_ for 3.5 hr in the presence of Brefeldin A (BD GolgiPlug), or alternatively, with eBioscience™ Cell Stimulation Cocktail plus protein transport inhibitors (Thermo Fisher Scientific, 00-4975-93). After stimulation, cells were surface stained as mentioned above. Then, cells were fixed and permeabilized using BD Cytofix/Cytoperm fixation/permeabilization kit (BD) followed by intracellular staining of cytokines (IFN-γ, IL-2 and TNF-α) as indicated by the manufacturer protocol.

#### 4.2.8 Tetramer staining

For OVA-specific CD8+ T cell quantification cells were incubated with PE H2-K^b^-OVA (SIINFEKL) tetramers (MBL) at room temperature for 30 min protected from light, followed by surface staining and FACS analysis.

#### 4.2.9 t-SNE and clustering

For tSNE visualization of tumor immune infiltrate a multicolor flow cytometry panel was used including 19 parameters (FSC, SSC, Viability, CD45, VenusFP, XCR1, CD4, NK1.1, CD26, F4/80, Ly6G, MHCII, CD24, CD3e, Ly6C, CD8a, CD11c, CD11b, CD19). Cells were compensated for spillover between channels and pre-gated on CD45+ Live singlets using FlowJo. Flowjo workspace was imported into the R environment using CytoML v2.4.0, FlowWokspace v4.4.0 and FlowCore v2.4.0 packages (63–65). The intensity values of marker expression were then biexp-transformed via the flowjo_biexp_trans function of FlowWorkspace using parameters ChannelRange=4096, maxValue=262144, pos=4.5, neg=0 and widthBasis=-10. Subsequently 5.000 cell events from each mouse (4 WT and 4 ERAI) were randomly sampled and combined for a total of 40.000 single cells. Sampled data was min-max normalized, and subjected to dimensionality reduction by Barnes-Hutts implementation of t-Distributed Stochastic Neighbor Embedding (tSNE) using RtSNE v0.15 package (66). Thirteen parameters were used for tSNE construction (XCR1, CD4, NK1.1, CD26, F4/80, Ly6G, MHCII, CD24, CD3e, Ly6C, CD8a, CD11c, CD11b and CD19) and the parameters were set to iterations=1000 and perplexity =30. After dimensionality reduction, automatic clustering was performed using density based spatial clustering (DBSCAN) using DBSCAN v1.1.8 package (67). Dotplot for marker expression among clusters and Violin plots for VenusFP were then generated using ggplot2 v3.3.5 package (68).

#### 4.2.10 *In vivo* T cell proliferation assay

LN cells from pmel-1 TCR transgenic mice were isolated and enriched for CD8+ T cells by magnetic negative selection using CD8+ T cell isolation kit (MACS, Miltenyi). Enriched CD8+ T cells were surface stained and naïve CD8+ T cells were purified by cell sorting (CD8a+, CD62L high, CD44low, CD25 neg). After sorting, naïve CD8+ T cells were labeled with Cell Trace Violet (CTV, Invitrogen). 1x10^6^ naïve CD8+ T cells were adoptively transferred into B16-F10 tumor-bearing mice at day 7 after tumor challenge. *In vivo* proliferation and CD44/CD25 expression of transferred T cells was analyzed by FACS in tumor draining lymph nodes 4 days after adoptive transfer.

#### 4.2.11 RNA-seq

Cell suspensions from tumor tissue pooled from 2-4 B16 bearing mice were enriched in immune cells by positive selection with CD45+ biotin magnetic beads (MACS, Miltenyi). Enriched cells were surface stained and 5-20 x10^3^ intratumoral cDC1s were sorted directly in RLT lysis buffer (Qiagen) containing 2-mercaptoethanol. Immediately after sorting, collected cells were homogenized through vortex and frozen on dry ice before storage at -80°C. Total RNA was extracted with RNAeasy Plus Micro kit (Qiagen). RNA sequencing was performed at VIB Nucleomics Core using SMART-seq v4 pre-amplification followed by single-end sequencing on Illumina NextSeq500. Preprocessing of the RNA-seq data was performed by Trimmomatic v0.39 and quality control by FastQC v0.11.8. Mapping to the reference mouse genome was performed by STAR v2.7.3a and HTSeqCount v0.11.2 was used for counting. Limma v3.42.2 (69) was used to normalize the data. Genes which did not meet the requirement of a count per million (cpm) value larger than 1 in at least 4 samples were filtered. This resulted in an expression table containing 11066 genes. EdgeR v3.28.0 (70) was utilized to perform differential expression analysis. Benjamini-Hochberg correction was used to adjust the p-values for multiple testing. Differentially expressed genes were filtered as genes with a |FC| > 1.5 and adjusted p-value < 0.05 (**Supplementary Table 1**). Heatmaps were created using pheatmap v1.0.12 package (71) on log2 normalized and mean centered gene expression data.

#### 4.2.12 Gene set enrichment analysis

Over-Representation Analysis (ORA) and Gene Set Enrichment Analysis (GSEA) were performed using *ClusterProfiler* v4.0.5 package (72) in R and Gene Ontology (GO) knowledgebase gene sets. ORA results were considered significant when the q-value was below 0.01. GSEA was performed on pre-ranked mode using as rank metric the signed log10 transformed p-values derived from the differential expression analysis. GSEA was run using the GO : BP database or literature gene sets of Xbp1- and RIDD-targets (34) (**Supplementary Table 2**). Results were considered significant when the adjusted p-value was below 0.05. Normalized expression values of the genes of interest from the literature gene sets (e.g. XBP1-targets) were transformed to z-scores (z = (x - µ)/σ) and the average z-score for each group (genotype) was visualized with box plots.

### 4.3 Quantification and statistical analysis

No statistical methods were used to predetermine sample size. The experiments were not randomized, and the investigators were not blinded to allocation during experiments and outcome assessment. Statistical analysis was conducted using GraphPad Prism software (v9.1.2). Results are presented as mean ± SEM. Two groups were compared using two tailed t-test for normal distributed data (Shapiro-Wilk test) or using a non-parametric two-tailed Mann-Whitney test as indicated in figure legends. Multiple groups were compared using one-way ANOVA with Tukey post-test. A p-value < 0.05 was considered statistically significant.

### 4.4 Study approval

All animal procedures were approved and performed in accordance with institutional guidelines for animal care of the Fundación Ciencia y Vida, the Faculty of Medicine, University of Chile and the VIB site Ghent-Ghent University Faculty of Sciences and were approved by the local ethics committee.

## Data availability statement

The datasets presented in this study can be found in online repositories. The names of the repository/repositories and accession number(s) can be found below: https://www.ncbi.nlm.nih.gov/geo/, GEO: GSE195439.

## Ethics statement

All animal procedures were performed in accordance with institutional guidelines for animal care of the Fundación Ciencia y Vida, the Faculty of Medicine, University of Chile and the VIB site Ghent-Ghent University Faculty of Sciences and were approved by the local ethics committee.

## Author contributions

FF-S, MB, SJ and FO designed the research. FF-S, SR, EV, SG, CF, DFi did the experiments. FF-S, SR, SJ and FO analyzed the results. CD and CM helped with RNA-seq data analysis. DFe provided technical assistance and experimental expertise, TI and ÁL provided critical reagents. FF-S and FO wrote the manuscript. All authors contributed to the article and approved the submitted version.
