# Supplementary material for: Nuanced role for dendritic cell intrinsic IRE1 RNase in the regulation of antitumor adaptive immunity
Source: Front Immunol. 2023 Jun 6;14:1209588. doi: 10.3389/fimmu.2023.1209588 (PMC10279875; doi:10.3389/fimmu.2023.1209588)

Supplementary Material

Nuanced role for dendritic cell intrinsic IRE1 RNase in the regulation of antitumor adaptive immunity

Felipe Flores-Santibañez, Sofie Rennen, Dominique Fernandez, Clint De Nolf, Evelien Van De Velde, Sandra Gaete, Camila Fuentes, Carolina Moreno, Diego Figueroa, Álvaro Lladser, Takao Iwawaki, María Rosa Bono, Sophie Janssens* and Fabiola Osorio*

*** Correspondence:**

Fabiola Osorio
Immunology Program
Institute of Biomedical Sciences
Faculty of Medicine
Universidad de Chile
Av. Independencia 1027
Postal code: 8380453
Santiago
Chile
Phone: +56-2-29789503
Email: fabiolaosorio@med.uchile.cl

Sophie Janssens
Laboratory for Endoplasmic Reticulum Stress and Inflammation
VIB Center for Inflammation Research,
Technologiepark 71
B-9052 Zwijnaarde
Ghent
Belgium
Email: sophie.janssens@irc.vib-ugent.be

1 Reagents and tools table

REAGENT or RESOURCE	SOURCE	IDENTIFIER
Antibodies		
B220 BV510	Biolegend	clone RA3-6B2; #cat 103222; RRID:AB_313005
B220 PE-Cy5	Biolegend	clone RA3-6B2; #cat 103209; RRID:AB_312994
CD11b BV785	Biolegend	clone M1/70; #cat 101243; RRID:AB_2561373
CD11c APC	Thermo Fisher Scientific	clone N418; #cat 17-0114-82; RRID:AB_469346
CD11c BV711	Biolegend	clone N418; #cat 117349; RRID:AB_2563905
CD16/32 (FcBlock)	Biolegend	clone 93; #cat 101302; RRID:AB_312801
CD18 PE	BD Biosciences	clone C71/16; #cat 553293; RRID:AB_394762
CD19 BUV737	BD Biosciences	clone 1D3; #cat 564296; RRID:AB_2716855
CD25 PE	Tonbo Bioscience	clone PC61.5; #cat 50-0251; RRID:AB_2621757
CD26 PE-Cy7	Biolegend	clone H194-112; #cat 137809; RRID:AB_2564311
CD274 (PD-L1) PE	Thermo Fisher Scientific	clone MIH5; #cat 12-5982-82; RRID:AB_466089
CD3 BV711	Biolegend	clone 17A2; #cat 100241; RRID:AB_2563945
CD39 SuperBright600	Thermo Fisher Scientific	clone 24DMS1; #cat 63-0391-82; RRID:AB_2717037
CD3ε BV510	Biolegend	clone 145-2C11; #cat 100353; RRID:AB_2565879
CD3ε PE-Cy5	Biolegend	clone 145-2C11; #cat 100309; RRID:AB_312674

CD4 APC-Cy7	Tonbo Bioscience	clone GK1.5; #cat 25-0041; RRID:AB_2904484
CD4 PE/Dazzle 594	Biolegend	clone GK1.5; #cat 100455; RRID:AB_2565844
CD40 APC	Biolegend	clone 3/23.; #cat 124611; RRID:AB_1134081
CD44 PerCP	Biolegend	clone IM7; #cat 103036; RRID:AB_10645506
CD44 FITC	Biolegend	clone IM7; #cat 103006; RRID:AB_312957
CD45 BV785	Biolegend	clone 30-F11 ; #cat 103149; RRID:AB_2564590
CD45 Alexa Fluor 700	Biolegend	clone 30-F11; #cat 103127; RRID:AB_493714
CD45 BUV395	BD Biosciences	clone 30-F11; #cat 564279; RRID:AB_2651134
CD45 BV650	Biolegend	clone 30-F11; #cat 103151; RRID:AB_2565884
CD45.1 APC-Cy7	Biolegend	clone A20; #cat 110715; RRID:AB_313504
CD62L PE-Cy7	Biolegend	clone MEL-14 ; #cat 104418; RRID:AB_313103
CD64 BV711	Biolegend	clone X54-5/7.1 ; #cat 139311; RRID:AB_2563846
CD64 PE/Dazzle 594	Biolegend	clone X54-5/7.1; #cat 139319; RRID:AB_2566558
CD64 PerCP-Cy5.5	Biolegend	clone X54-5/7.1; #cat 139307; RRID:AB_2561962
CD8a APC-Cy7	Tonbo Bioscience	clone 53-6.7; #cat 25-0081; RRID:AB_2621623
CD8a BV650	Biolegend	clone 53-6.7; #cat 100742; RRID:AB_2563056
CD8a eFluor 450	Thermo Fisher Scientific	clone 53-6.7; #cat 48-0081-80; RRID:AB_1272235

F4/80 Biotin	Biolegend	clone BM8; #cat 123105; RRID:AB_893499
FOXP3 PE-Cy7	Thermo Fisher Scientific	clone FJK-16s; #cat 25-5773-82; RRID:AB_891552
Granzyme B PE Texas RED	Thermo Fisher Scientific	clone GB11; #cat GRB17; RRID:AB_2536540
I-A/I-E (MHC-II) APC-Cy7	Biolegend	clone M5/114.15.2; #cat 107627; RRID:AB_1659252
IFN-γ PE	Thermo Fisher Scientific	clone XMG1.2; #cat 12-7311-82; RRID:AB_466193
IL-12p40 eFluor660	Thermo Fisher Scientific	clone C17.8; #cat 50-7123-80; RRID:AB_11218284
IL-2 PE-Cy7	Thermo Fisher Scientific	clone JES6-5H4; #cat 25-7021-82; RRID:AB_1235004
Ly6C BV605	Biolegend	clone HK1.4; #cat 128035; RRID:AB_2562352
Ly6G Alexa Fluor 700	Biolegend	clone 1A8; #cat 127622; RRID:AB_10643269
NK1.1 PerCP-Cy5.5	Biolegend	clone PK136; #cat 108727; RRID:AB_2132706
PD1 PE	Biolegend	clone 29F.1A12; #cat 135206; RRID:AB_1877231
PD1 BV421	Biolegend	clone 29F.1A12; #cat 135217; RRID:AB_10900085
TCF1 Alexa Fluor 488	Cell Signaling	clone C63D9; #cat CS.6444S; RRID:AB_2797627
TIM3 PECy7	Biolegend	clone RMT3-23; #cat 119716; RRID:AB_2571933
TNF-α APC	Biolegend	clone MP6-XT22; #cat 506307; RRID:AB_315428
TOX APC	Miltenyi Biotec	clone REA473; #cat 130-118-335; RRID:AB_2751485

XCR1 BV650	Biolegend	clone ZET; #cat 148220; RRID:AB_2566410
XCR1 PE	Biolegend	clone ZET; #cat 148204; RRID:AB_2563843
CD16/32 (FcBlock)	Bioceros	Clone 2.4G2
CD103 BUV395	BD Biosciences	Clone M290; 740238
CD45 BUV496	BD Biosciences	Clone 30-F11; 749889
CD11c BUV737	BD Biosciences	Clone HL3; 612796
MHCII BUV805	BD Biosciences	Clone M5/114.15.2; 748844
CD26 BV421	BD Biosciences	Clone H194-112; 740021
CD3e BV605	Biolegend	Clone 145-2c11; 100351
CD19 BV605	Biolegend	Clone 6D5; 115540
XCR1 BV650	Biolegend	Clone ZET; 148220
CD64 BV711	Biolegend	Clone X54-5/7.1; 139311
F4/80 BV786	Biolegend	Clone BM8; 123141
Ly6C PerCP-Cy5-5	Themro Fisher Scientific	Clone HK1.4; 45-5932-82
CD80 PE	BD Biosciences	Clone 16-10A1; 553769
Streptavidin PE-CF594	BD Biosciences	#cat 562284
CD161 PE-Cy5	Biolegend	Clone PK136; 108716
CD172a PE-Cy7	Biolegend	Clone P84; 144008
CD86 APC	Biolegend	Clone GL1; 105012
CD11b AF700	BD Biosciences	Clone M1/70; 564985
CCR7 biotin	Thermo Fisher Scientific	Clone 4B12; 13-1971-85
IL-12p40 PE	Thermo Fisher Scienific	Clone C17.8; 12-7123-81
CD40 APC	Biolegend	Clone 3/23; 124612
Foxp3 eFluor450	Thermo Fisher Scientific	Clone FJK-16s; 48-5773-82
CD11b BV605	BD Biosciences	Clone M1/70; 563015
CD44 BV650	Biolegend	Clone IM7; 103049
TCRb BV786	Biolegend	Clone H57-597; 109249
CD8a PerCP-Cy5-5	Thermo Fisher Scientific	Clone 53-6,7; 45-0081-82
PD-1 PE	Biolegend	Clone 29F.1A12; 135206
Granzyme B PE-TR	Thermo Fisher Scientific	Clone GB11; GRB17

CD45 APC-R700	BD Biosciences	Clone 30-F11; 565478
CD4 APC-Cy7	Tonbo Biosciences	Clone GK1.5; 25-0041-U100
CD161 PE-TR	BD Biosciences	Clone PK136; 562864
CD3 biotin	Thermo Fisher Scientific	Clone 145-2C11; 13-0031-85
CD19 biotin	Thermo Fisher Scientific	Clone eBio1D3(1D3); 13-0193-85
CD64 biotin	Biolegend	Clone X54-5/7.1; 139318
CD161 biotin	Thermo Fisher Scientific	Clone PK136; 13-5941-85
TER119 biotin	Thermo Fisher Scientific	Clone TER-119; 13-5921-82
Ly6G biotin	Biolegend	Clone 1A8, 127604
CD11c eFluor660	Thermo Fisher Scientific	Clone N418; 50-0114-82
MHCII APC-eFluor780	Thermo Fisher Scientific	Clone M5/114.15.2; 47-5321-82
CD64 BV421	Biolegend	Clone X54-5/7.1; 139309
Bacterial and virus strains		
Biological samples		
Mouse tumor draining lymph nodes (inguinal)	This paper	N/A
Mouse tumor	This paper	N/A
Mouse spleen	This paper	N/A
Mouse bone marrow	This paper	N/A
Chemicals, peptides, and recombinant proteins		
Recombinant Human Flt3-Ligand (FLT3-L)	Peprotech	#cat 300-19
Brefeldin A	Cayman Chemical	#cat 11861
PMA	Sigma	#cat P8139
Ionomycin	Sigma	#cat I0634
Critical commercial assays		
CD45 MicroBeads, mouse	Miltenyi	RRID:AB_2877061
CD8a+ T Cell Isolation Kit, mouse	Miltenyi	#cat 130-104-075
LS columns	Miltenyi	#cat 130-042-401
BD Cytofix/Cytoperm with GolgiPlug	BD Biosciences	RRID:AB_2869013
Foxp3 / Transcription Factor Staining Buffer Set	eBioscience	#cat 00-5523-00
RNeasy Plus Micro Kit	Qiagen	#cat 74034
Ovation PicoSL WTA System V2 kit	TECAN	# cat 3312-48
iTAg Tetramer/PE - H-2 Kb OVA (SIINFEKL)	MBL	#cat T03000
MinElute Reaction Cleanup kit	Qiagen	# cat 28206
CellTrace™ Violet Cell Proliferation Kit	Thermo Fisher Scientific	#cat C34557
Zombie UV™ Fixable Viability Kit	Biolegend	#cat 423107

Zombie Green™ Fixable Viability Kit	Biolegend	#cat 423111
LIVE/DEAD™ Fixable Aqua Dead Cell Stain Kit	Thermo Fisher Scientific	#cat L34966
LIVE/DEAD™ Fixable Near-IR Dead Cell Stain Kit	Thermo Fisher Scientific	#cat L34975
eBioscience Fixable Viability dye eFluorTM 780	Thermo Fisher Scientific	#cat 65-0865-14
eBioscience Fixable Viability dye eFluorM 506	Thermo Fisher Scientific	#cat 65-0866-18
GoTaq G2 Green Master Mix 2X	Promega	# cat M7823
SensiFAST SYBR no-ROX mix	Bioline	#cat BIO-86020
UltraComp eBeads™ Plus Compensation Beads	Thermo Fisher Scientific	#cat 01-3333-42
Cell Stimulation Cocktail 500x	Thermo Fisher Scientific	#cat 00-4975-93
MagniSort™ Streptavidin Negative Selection Beads	Thermo Fisher Scientific	#cat MSNB-6002-74
Deposited data		
RNA-seq data	This paper	GEO: GSE195439
Experimental models: Cell lines		
B16-F10	ATCC	RRID:CVCL_0159
B78ChOVA: B78-mCherry OVA	(Broz et al., 2014)	N/A
B16ChOVA: B16-F10-mCherry OVA	(Schuijs et al., 2020)	N/A
MC38	(Corbett et al., 1975)	RRID:CVCL_B288
OP9-DL1	(Schmitt and Zúñiga-Pflücker, 2002)	RRID:CVCL_B218
Experimental models: Organisms/strains		
ERAI: C57BL/6J-Tg(CAG-XBP1*/venus)#Miur/MiurRbrc	(Iwawaki et al., 2004)	RRID:IMSR_RBRC01099
Itgax-Cre: B6.Cg-Tg(Itgax-cre)1-1Reiz/J	The Jackson Laboratory	RRID:IMSR_JAX:008068
Xcr1-Cre: B6. XCR1tm3/mtfp CIPHE	(Mattiuz et al., 2018)	N/A
IRE1 fl/fl: B6;129S4-Ern1<tm2.1Tiw>	(Iwawaki et al., 2009)	RRID:IMSR_RBRC05515
XBP1 fl/fl: Xbp1tm2Glm	(Lee et al., 2008)	RRID:MGI:6273536
Pmel-1: B6.Cg-Thy1a/Cy Tg(TcraTcrb)8Rest/J	The Jackson Laboratory	RRID:IMSR_JAX:005023
Oligonucleotides		
Primer: Splicing assay Xbp1 Forward: ACACGCTTGGGAATGGACAC	(Osorio et al., 2014)	N/A
Primer: Splicing assay Xbp1 Reverse: CCATGGGAAGATGTTCTGGG	(Osorio et al., 2014)	N/A
Primer: Actb Forward: GTGACGTTGACATCCGTAAAGA	This Paper	N/A
Primer: Actb Reverse: GCCGGACTCATCGTACTCC	This Paper	N/A
Primer: Ern1 (exon 19-20) forward TGCTGAAACACCCCTTCTTC	(Osorio et al., 2014)	N/A
Primer: Ern1(exon 19-20) reverse GCCTCCTTTTCTATTCGGTCA	(Osorio et al., 2014)	N/A

Primer: Xbp1 (exon 2) forward CAGCAAGTGGTGGATTTGG	(Tavernier et al., 2017)	N/A
Primer: Xbp1 (exon 2) reverse CGTGAGTTTTCTCCCGTAAAAG	(Tavernier et al., 2017)	N/A
Primer: Ywhaz forward CTCTTGGCAGCTAATGGGCTT	This Paper	N/A
Primer: Ywhaz reverse GGAGGTGGCTGAGGATGGA	This Paper	N/A
Primer: Sdha forward TTTCAGAGACGGCCATGATCT	This Paper	N/A
Primer Sdha reverse TGGGAATCCCACCCATGTT	This Paper	N/A
Recombinant DNA		
Software and algorithms		
FlowJo™ Software v10	BD Biosciences	https://www.flowjo.com/solutions/flowjo; RRID:SCR_008520
GraphPad Prism v9	GraphPad	https://www.graphpad.com/; RRID:SCR_002798
Trimmomatic v0.39	Usadellab	RRID:SCR_011848
FastQC v0.11.8	Babraham Bioinformatics	RRID:SCR_014583
STAR v2.7.3a	STAR	RRID:SCR_004463
HTSeqCount v0.11.2	HTSeq	RRID:SCR_005514
R Studio v2021.09.0 Build 351	R Studio	https://rstudio.com/; RRID:SCR_000432
R v4.1.1	R Core Team (2020)	http://www.r-project.org/; RRID:SCR_001905
R package: CytoML v2.4.0	(Finak et al., 2018)	https://bioconductor.org/packages/release/bioc/html/CytoML.html
R package: FlowWorkspace v4.4.0	(Finak and Jiang, 2021)	RRID:SCR_001155
R package: FlowCore v2.4.0	(Ellis et al., 2021)	RRID:SCR_002205
R package: Rtsne v0.15	(Krijthe, 2015)	RRID:SCR_016342
R package: DBSCAN v1.1.8	(Hahsler et al., 2019)	https://cran.r-project.org/web/packages/dbscan/index.html
R package: Limma v3.42.2	(Ritchie et al., 2015)	RRID:SCR_010943
R package: EdgeR v3.28.0	(Robinson et al., 2010)	RRID:SCR_012802
R package: pheatmap v1.0.12	(Kolde, 2019)	RRID:SCR_016418
R package: ggplot2 v3.3.5	(Wickham, 2016)	RRID:SCR_014601
R package: ClusterProfiler v4.0.5	(Wu et al., 2021)	RRID:SCR_016884

qBase+ v3.2	Biogazelle	http://www.biogazelle.com/qbaseplus; RRID:SCR_003370
Other		

2 Supplementary Figure Legends

Supplementary Figure 1. Immune analysis of melanoma tumors derived from ERAI mice.

(A) t-SNE map as in figure 1A. Color gradient shows the expression of the indicated marker**. (B)** Dot plot showing marker expression across the different cell clusters identified as in figure 1A. Size of the dots represent the % of cells positive for that marker, while the color represents the mean fluorescence intensity. **(C)** Background signal from non-transgenic mice (WT) across the different cell clusters identified in figure 1A. Median fluorescence intensity is depicted with a "+" inside each violin plot. **(D)** Related to figure 1D. Histograms of VenusFP signal from manually gated immune populations from B16-F10-bearing WT and ERAI mice. **(E)** Quantification of VenusFP signal from manually gated immune populations from MC38 bearing ERAI mice. **** $p < 0.0001$, ANOVA and Tukey post-test. n=5 ERAI mice, representative of two independent experiments. **(F)** Quantification of VenusFP signal from tumor and tumor draining lymph node migratory (mig) and resident (res) cDCs from tumor-bearing ERAI mice, or LN-resident cDCs from tumor-free ERAI mice. * $p<0.05$, **** $p<0.0001$, ANOVA and Tukey post-test. n=3 for tumor-free mice, n=5 for tumor-bearing mice.

Supplementary Figure 2. Gating strategy for tumor associated cDCs.

(A) Gating strategy for identification of immune infiltrated populations in tumors. Representative plots from B16-F10 melanoma tumor. **(B)** Gating strategy for identification of migratory (mig) and resident (res) cDC1s and cDC2s in tumor draining lymph node. Representative plots from B16-F10 melanoma.

Supplementary Figure 3. Gene Set Enrichment Analysis of XBP1-deficient and IRE1/XBP1-deficient tumor cDC1s.

(A) Related to figure 2C. Upon Cre mediated recombination in XBP1fl/fl mice, a premature stop codon is introduced in the Xbp1 mRNA sequence, preventing the translation of a functional XBP1s protein. However, IRE1 RNase activity can still be monitored by determining Xbp1 mRNA splicing ratio. Scheme depicts *LoxP* sites and IRE1 splicing sites at *Xbp1* locus. Primers for *Xbp1* splicing assay are also shown. **(B)** Related to figure 2D. Over representation analysis of DEGs over the Gene Ontology (GO) database. **(C)** Related to figure 2E. Gene Set Enrichment Analysis (GSEA) of WT vs XBP1$^{\Delta DC}$ or WT vs XBP1$^{\Delta DC}$IRE1^{truncDC} cDC1s using XBP1s- and RIDD-target gene sets from literature (37). **(D)** Related to figure 2F. GSEA of the "triglyceride biosynthetic process" gene set (GO: 0019432) in WT vs XBP1$^{\Delta DC}$ cDC1s (left) or WT vs XBP1$^{\Delta DC}$IRE1^{truncDC} cDC1s (right) showing not statistically significant enrichment (q-value > 0.05).

Supplementary Figure 4. $CD4^+$ T cells in B78ChOVA-bearing $XBP1^{\Delta DC}IRE1^{truncDC}$ mice.

Related to figure 3B-C. $XBP1^{WT}IRE1^{WT}$ and $XBP1^{\Delta DC}IRE1^{truncDC}$ mice were implanted with B78ChOVA cells. **(A)** Frequencies of intratumoral $CD4^+$ T cells. **(B)** Frequencies of cytokine producing intratumoral $CD4^+$ T cells after *ex vivo* stimulation with PMA/Ionomycin in the presence of BFA. **(A-B)** n=9 mice ($XBP1^{WT}IRE1^{WT}$) or 11 mice ($XBP1^{\Delta DC}IRE1^{truncDC}$), data pooled from three independent experiments, mean ± s.e.m.

Supplementary Figure 5. Tumor growth and T cell profiles of MC38-bearing $XBP1^{\Delta DC}$ mice.

$XBP1^{WT}$ and $XBP1^{\Delta DC}$ mice were implanted with MC38 cells. **(A)** Tumor growth curves monitored over a period of 15 days and boxplot of tumor volume at end point. n=12 ($XBP1^{WT}$) or n=13 ($XBP1^{\Delta DC}$) mice per group. Pooled data from two independent experiments. **(B-C)** $CD8^+$ T cell (B) and $CD4^+$ T cell (F) frequencies and profiles in MC38 bearing $XBP1^{WT}$ and $XBP1^{\Delta DC}$ mice. n=11 ($XBP1^{WT}$) or n=13 ($XBP1^{\Delta DC}$) mice per group, pooled data from two independent experiments, mean ± s.e.m.

Supplementary Figure 6. Immune cell profiling of B78ChOVA-bearing $XBP1^{\Delta DC}$ mice.

$XBP1^{WT}$ and $XBP1^{\Delta DC}$ mice were implanted with B78ChOVA cells. **(A)** Cell counts for tumor immune infiltrate ($CD45^+$). n=31 mice ($XBP1^{WT}$) or 29 mice ($XBP1^{\Delta DC}$), pooled data from 8 independent experiments, mean ± s.e.m (left). Summary of mean frequencies of tumor lymphoid and myeloid populations. n=3-12 mice per group (right). **(B)** Tumor $CD4^+$ T cell, Treg ($CD3^+CD4^+Foxp3^+$) and Tconv ($CD3^+CD4^+Foxp3^-$) frequencies, and $CD8^+$ T/Treg ratio. n=8 mice per group, pooled data from two independent experiments, mean ± s.e.m. **(C)** Frequencies of cytokine producing tumor $CD4^+$ T cells after *ex vivo* stimulation with PMA/Ionomycin in the presence of BFA. *$p < 0.05$, two-tailed Mann-Whitney test. n=18 mice ($XBP1^{WT}$) or 17 mice ($XBP1^{\Delta DC}$), pooled data from 4 independent experiments, mean ± s.e.m. **(D)** Related to figure 3H. Representative histograms of different markers associated with exhausted $CD8^+$ T cells. Gated on $CD3^+CD8^+PD\text{-}1^+TCF\text{-}1^+$ or $CD3^+CD8^+PD\text{-}1^+TIM\text{-}3^+$ as shown in figure 3H. **(E-F)** $H\text{-}2K^bOVA_{257\text{-}264}$ $Tetramer^+$ $CD8^+$ T cell frequencies in TdLN (E) or tumor (F). Fluorescence Minus One (FMO) and inguinal lymph nodes from tumor-free $XBP1^{WT}$ mice (no tumor) are shown as control. Gated on $CD3^+CD8^+$ T cells. n=4 (TdLN tumor-free), n=6 mice per group (TdLN $XBP1^{WT}$ and $XBP1^{\Delta DC}$) or n=8 mice per group (tumor $XBP1^{WT}$ and $XBP1^{\Delta DC}$), pooled data from two independent experiments, mean ± s.e.m. **(G)** PD-L1 expression in tumor cDC1, cDC2 and TAM. n=4 mice per group, representative data from two independent experiments.

Supplementary Figure 7. *In vitro* generated cDC1s from $XBP1^{\Delta DC}$ mice produce normal levels IL-12 in response B78ChOVA lysate.

Intracellular IL-12 expression of *in vitro* generated cDC1s (FLT3-L/OP9-DL1) (57) stimulated with B78ChOVA lysates. Gated on cDC1s ($MHC\text{-}II^+$ $CD11c^+$ $CD8^+$ $CD11b^-$). *$p < 0.05$, two-tailed Mann-

Whitney test. Each dot represents a biological replicate, n=5, data pooled from three independent experiments, mean ± s.e.m.

Supplementary Figure 8. Immune cell profiling of tumor-bearing XBP1$^{\Delta cDC1}$ and XBP1$^{\Delta cDC1}$IRE1$^{trunc\text{-}cDC1}$ mice.

(A-B) Frequencies of intratumoral cDC subsets from MC38-bearing XBP1^{WT}IRE1^{WT} and XBP1$^{\Delta cDC1}$IRE1$^{trunc\text{-}cDC1}$ mice (A) or XBP1^{WT} and XBP1$^{\Delta cDC1}$ mice (B). n=6 mice (XBP1^{WT}IRE1^{WT}), n=9 mice (XBP1$^{\Delta cDC1}$IRE1$^{trunc\text{-}cDC1}$), n=10 mice (XBP1^{WT}) or n=7 mice (XBP1$^{\Delta cDC1}$), pooled from 2 independent experiments, mean ± s.e.m. **(C-D)** Real time quantitative PCR (RT-qPCR) analysis of *Ern1* exon 19-20 and *Xbp1* exon 2 in cDC1s sorted from MC38-bearing XBP1^{WT}IRE1^{WT} and XBP1$^{\Delta cDC1}$IRE1$^{trunc\text{-}cDC1}$ mice (C) or XBP1^{WT} and XBP1$^{\Delta cDC1}$ mice (D). RNA expression was normalized to housekeeping genes *Ywhaz* and *Sdha*. n=4 mice (XBP1^{WT}IRE1^{WT}), n=6 mice (XBP1$^{\Delta cDC1}$IRE1$^{trunc\text{-}cDC1}$), n=4 mice (XBP1^{WT}) or n=2 mice (XBP1$^{\Delta cDC1}$), *** p<0.001, *p<0.05, t-test. **(E)** CD4^{+} T cells frequencies and profiles from B16ChOVA-bearing (top) or MC38-bearing (bottom) XBP1^{WT}IRE1^{WT} and XBP1$^{\Delta cDC1}$IRE1$^{trunc\text{-}cDC1}$ mice. n=4-8 mice (XBP1^{WT}IRE1^{WT}) or n=7-11 mice (XBP1$^{\Delta cDC1}$IRE1$^{trunc\text{-}cDC1}$), data pooled from two independent experiments, mean ± s.e.m. **(F)** CD4^{+} T cells frequencies and profiles from B16ChOVA-bearing (top) or MC38-bearing (bottom) XBP1^{WT} and XBP1$^{\Delta cDC1}$ mice. n=4-10 mice (XBP1^{WT}) or n=8-11 mice (XBP1$^{\Delta cDC1}$), data pooled from two independent experiments, mean ± s.e.m. **(G)** PCR analysis of *Xbp1* splicing in intratumoral cDC1s isolated from MC38-bearing XBP1^{WT}IRE1^{WT} and XBP1$^{\Delta cDC1}$/IRE1$^{trunc\text{-}cDC1}$ mice (top) or XBP1^{WT} and XBP1$^{\Delta cDC1}$ mice (bottom). The lanes represent different mice. Xbp1u: *Xbp1* unspliced; Xbp1s: *Xbp1* spliced; Actb: beta actin. **(H)** CD11c expression by intratumoral cDC1s from MC38-bearing XBP1^{WT}IRE1^{WT} and XBP1$^{\Delta cDC1}$IRE1$^{trunc\text{-}cDC1}$ mice (top) and XBP1^{WT} and XBP1$^{\Delta cDC1}$ mice (bottom). n=4-5 mice per group, representative of two independent experiments, mean ± s.e.m. * p<0.05, Mann-Whitney test.

3 Supplementary Figures

Supplementary Figure 1

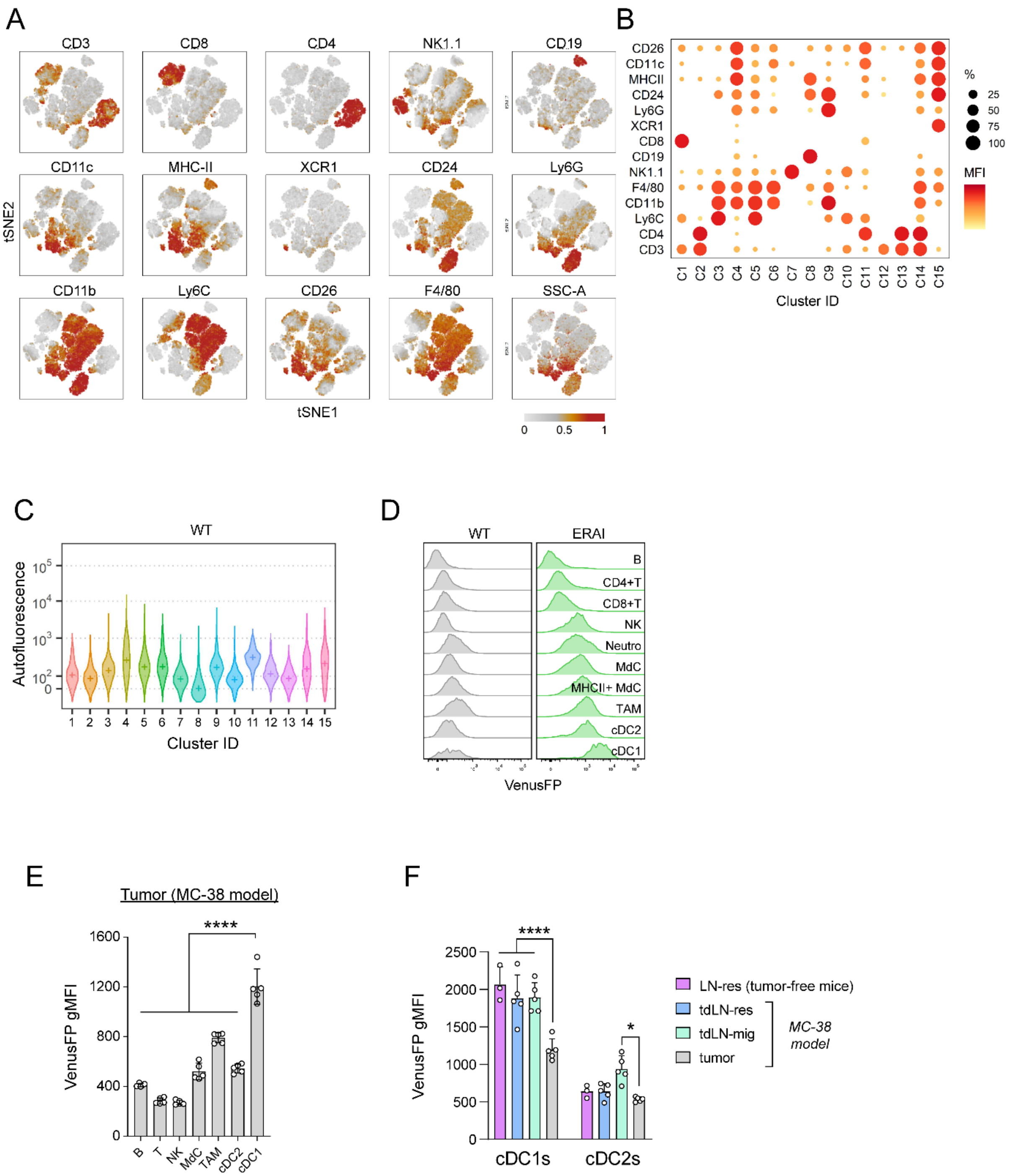

Supplementary Figure 2

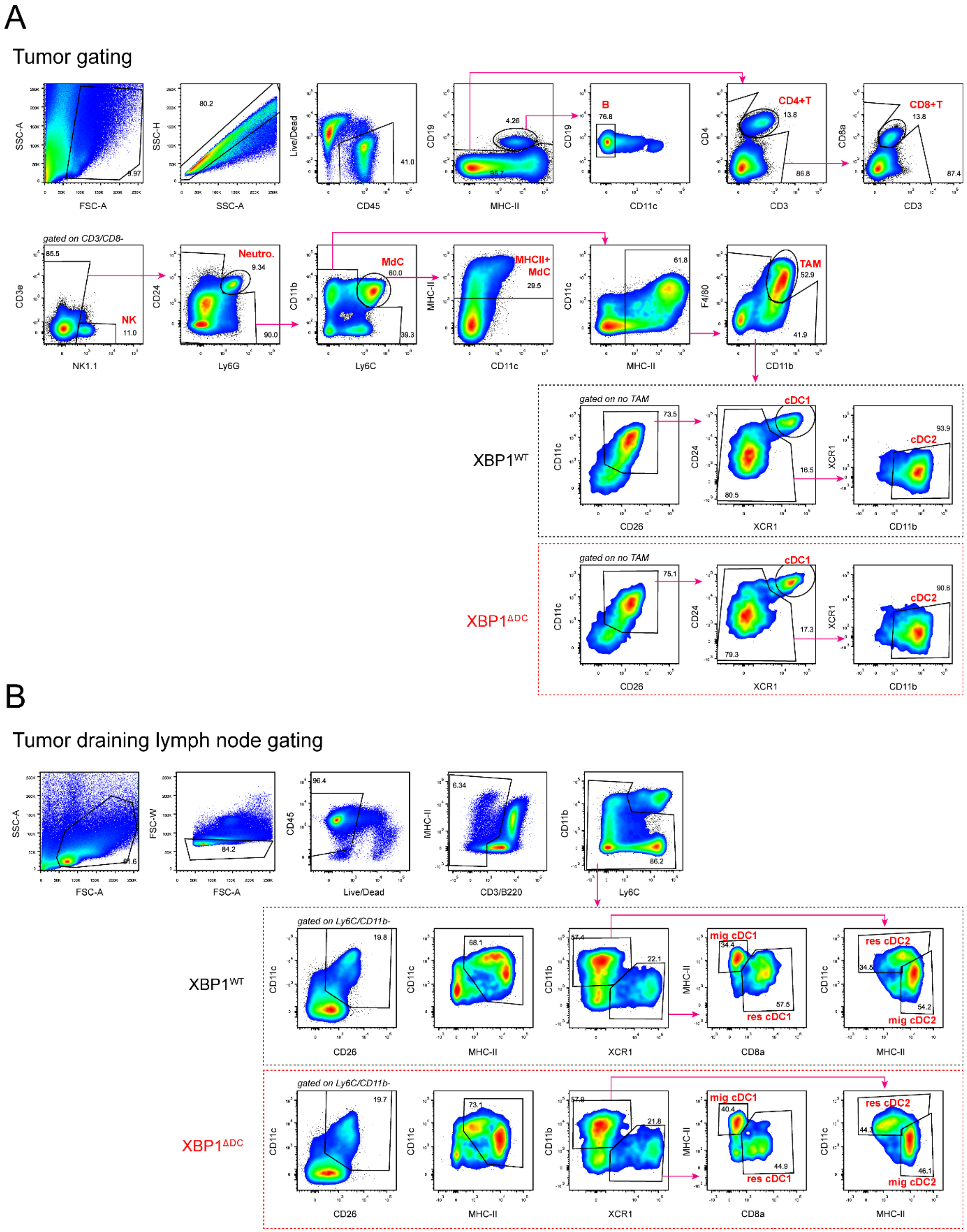

Supplementary Figure 3

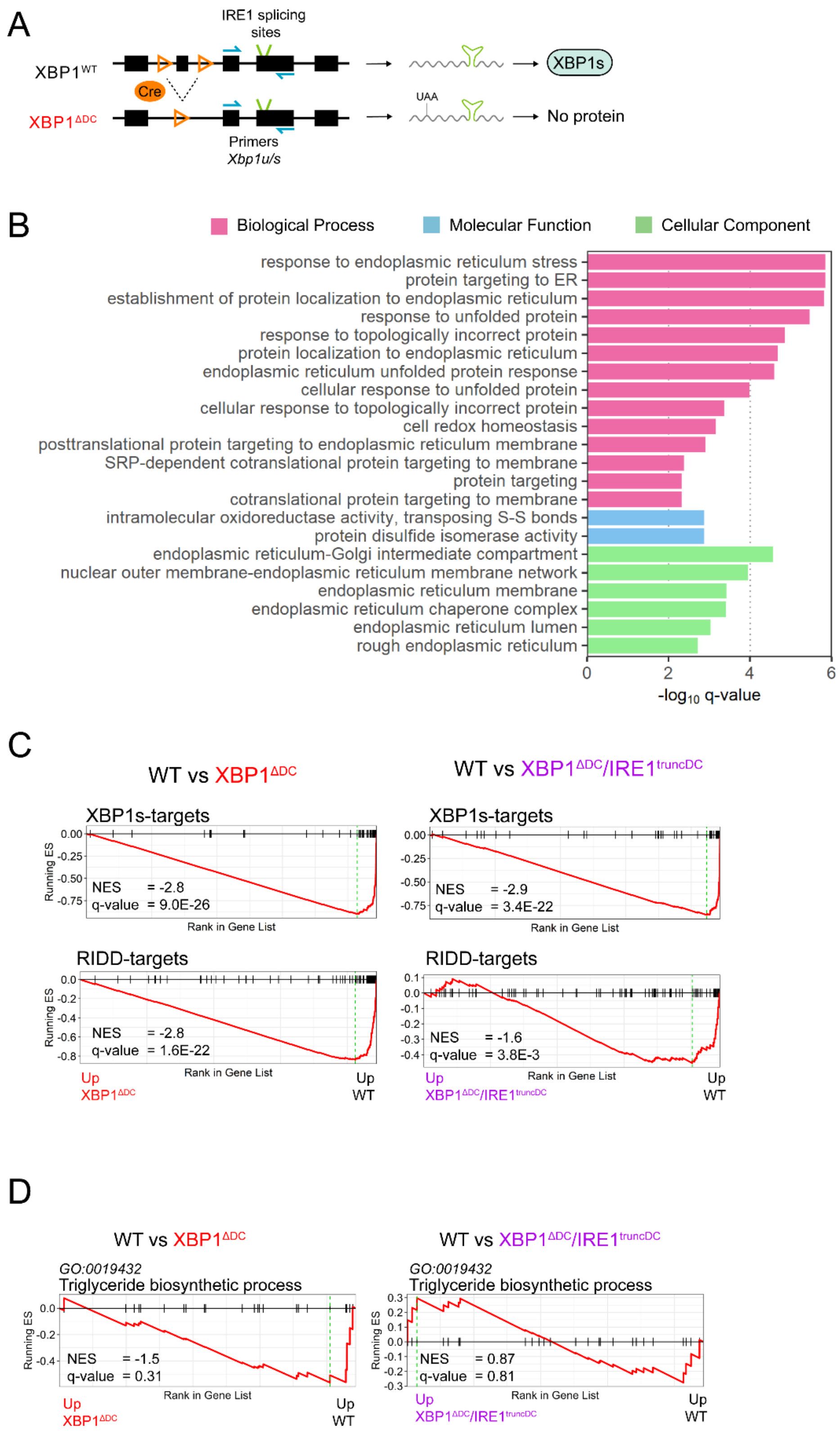

Supplementary Figure 4

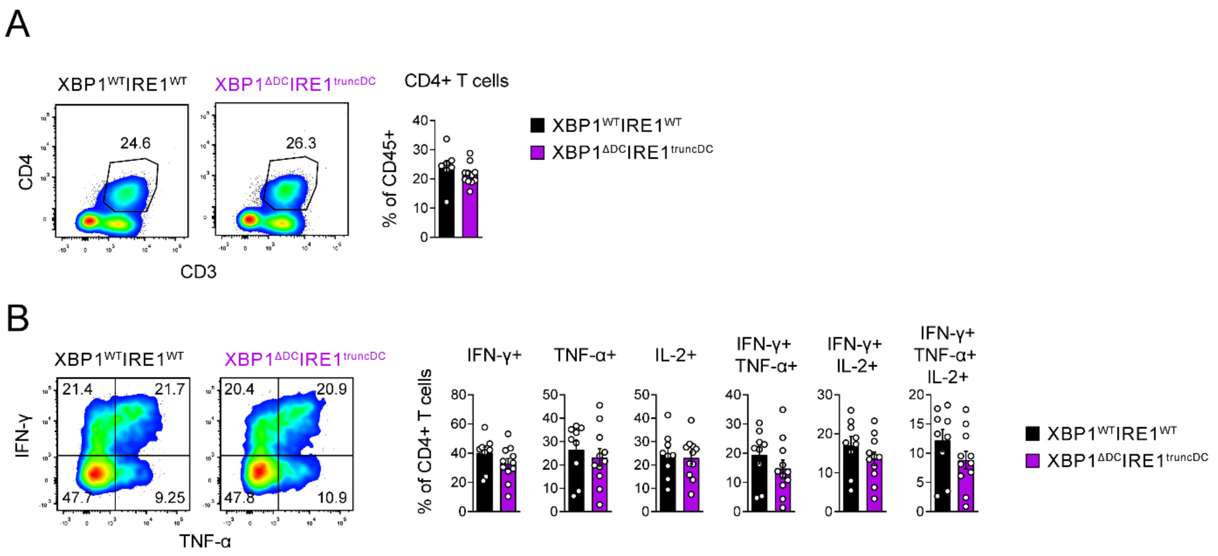

Supplementary Figure 5

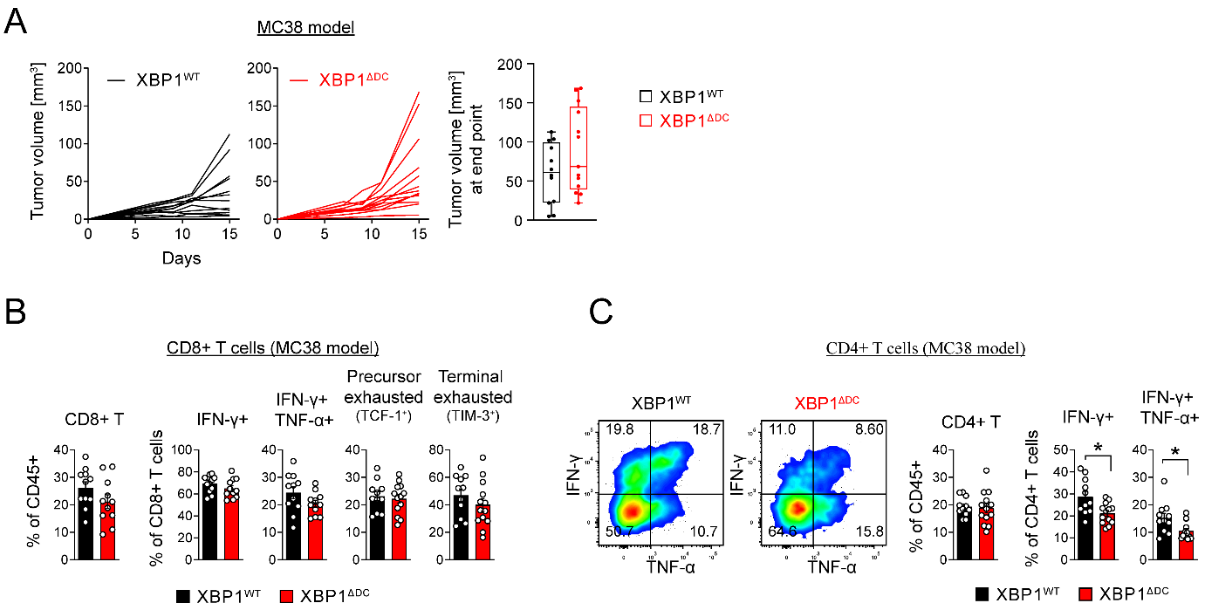

Supplementary Figure 6

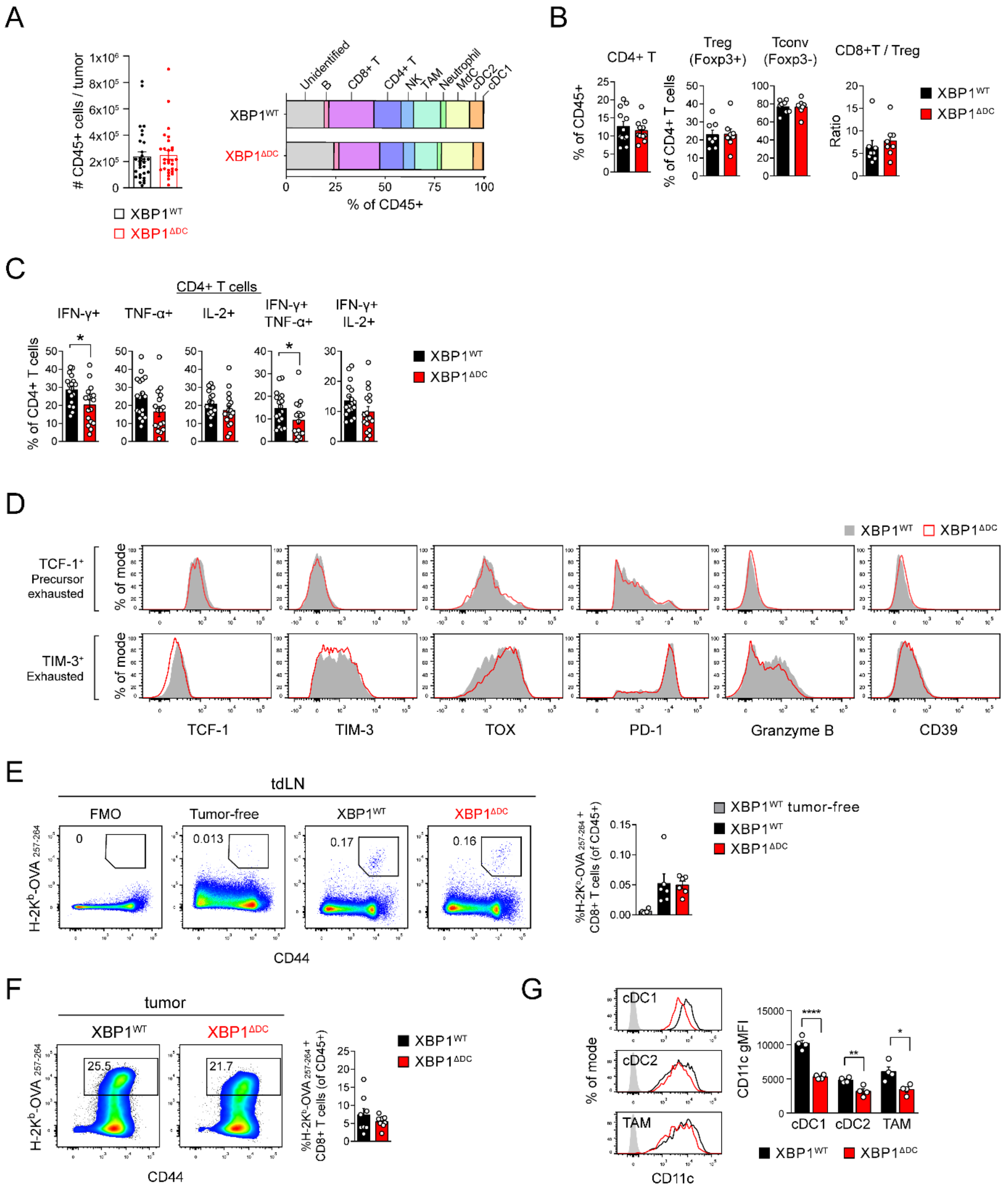

Supplementary Figure 7

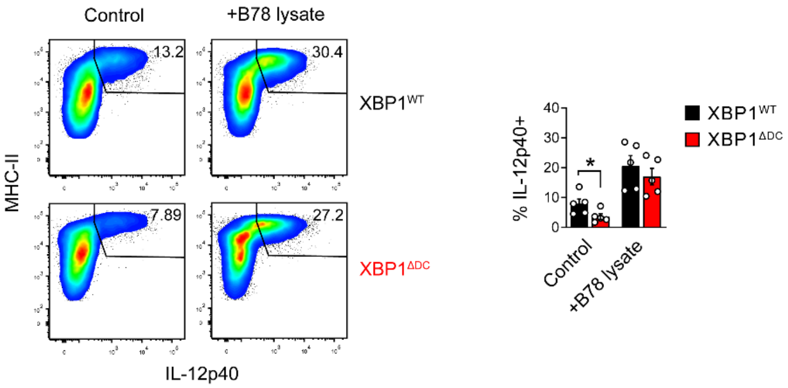

Supplementary Figure 8

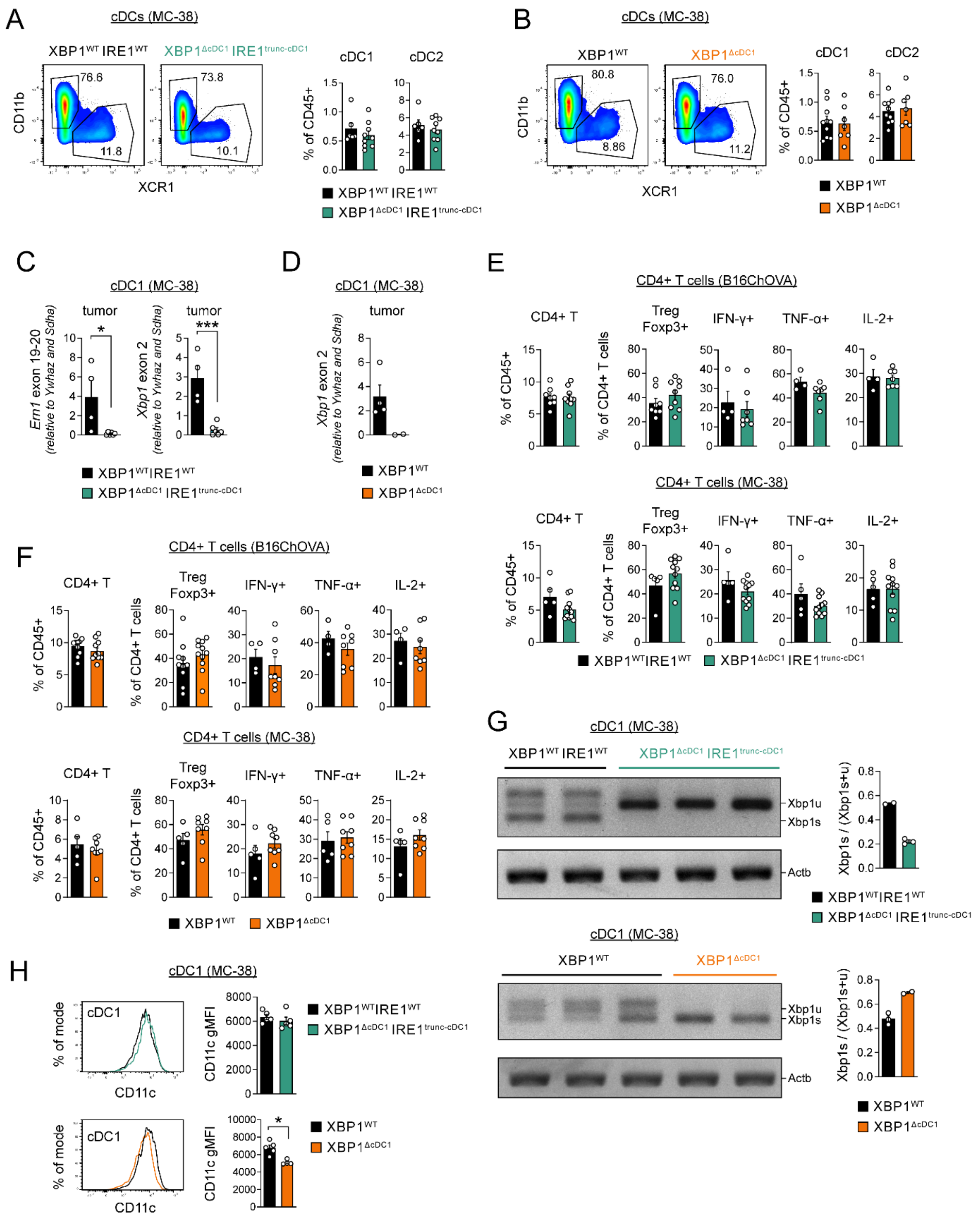

Supplement: Supplementary file 1 [file DataSheet_1.pdf]
